# Synaptic memory and CaMKII

**DOI:** 10.1152/physrev.00034.2022

**Published:** 2023-06-08

**Authors:** Roger A. Nicoll, Howard Schulman

**Affiliations:** ^1^Department of Cellular and Molecular Pharmacology, University of California at San Francisco, San Francisco, California, United States; ^2^Department of Neurobiology, Stanford University School of Medicine, Stanford, California, United States; ^3^Panorama Research Institute, Sunnyvale, California, United States

**Keywords:** AMPA receptor, calcium, CaMKII, long-term potentiation, NMDA receptor

## Abstract

Ca^2+^/calmodulin-dependent protein kinase II (CaMKII) and long-term potentiation (LTP) were discovered within a decade of each other and have been inextricably intertwined ever since. However, like many marriages, it has had its up and downs. Based on the unique biochemical properties of CaMKII, it was proposed as a memory molecule before any physiological linkage was made to LTP. However, as reviewed here, the convincing linkage of CaMKII to synaptic physiology and behavior took many decades. New technologies were critical in this journey, including in vitro brain slices, mouse genetics, single-cell molecular genetics, pharmacological reagents, protein structure, and two-photon microscopy, as were new investigators attracted by the exciting challenge. This review tracks this journey and assesses the state of this marriage 40 years on. The collective literature impels us to propose a relatively simple model for synaptic memory involving the following steps that drive the process: *1*) Ca^2+^ entry through *N*-methyl-d-aspartate (NMDA) receptors activates CaMKII. *2*) CaMKII undergoes autophosphorylation resulting in constitutive, Ca^2+^-independent activity and exposure of a binding site for the NMDA receptor subunit GluN2B. *3*) Active CaMKII translocates to the postsynaptic density (PSD) and binds to the cytoplasmic C-tail of GluN2B. *4*) The CaMKII-GluN2B complex initiates a structural rearrangement of the PSD that may involve liquid-liquid phase separation. *5*) This rearrangement involves the PSD-95 scaffolding protein, α-amino-3-hydroxy-5-methyl-4-isoxazolepropionic acid receptors (AMPARs), and their transmembrane AMPAR-regulatory protein (TARP) auxiliary subunits, resulting in an accumulation of AMPARs in the PSD that underlies synaptic potentiation. *6*) The stability of the modified PSD is maintained by the stability of the CaMKII-GluN2B complex. *7*) By a process of subunit exchange or interholoenzyme phosphorylation CaMKII maintains synaptic potentiation in the face of CaMKII protein turnover. There are many other important proteins that participate in enlargement of the synaptic spine or modulation of the steps that drive and maintain the potentiation. In this review we critically discuss the data underlying each of the steps. As will become clear, some of these steps are more firmly grounded than others, and we provide suggestions as to how the evidence supporting these steps can be strengthened or, based on the new data, be replaced. Although the journey has been a long one, the prospect of having a detailed cellular and molecular understanding of learning and memory is at hand.

## Graphical abstract

**Figure:**
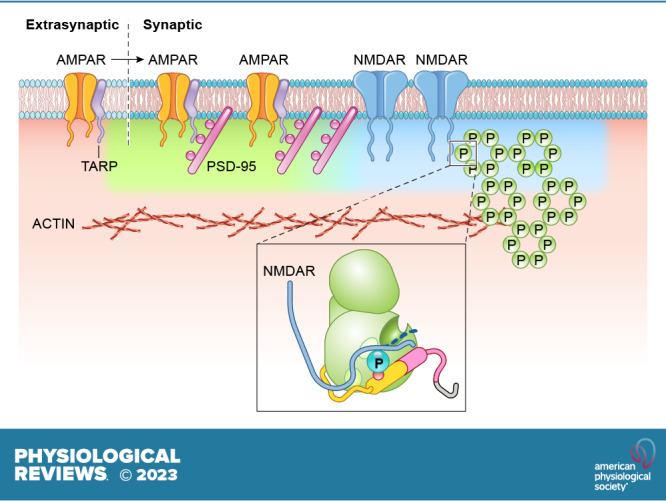

CLINICAL HIGHLIGHTS One of the most important functions of the brain is its ability to store information over long periods of time. It allows us as human beings to store internal representations of the external world and, based on this, plan and execute behaviors. The synaptic adaptations to episodic events and the lifetime accumulation of memories are critical in defining who we are as individuals. Mutations in two critical proteins, the Ca^2+^/calmodulin-dependent protein kinase II (CaMKII) and one of the glutamate receptors, GluN2B, have been found to underlie a range of intellectual disabilities and neurodevelopmental problems, such as autism. The role of these two proteins in hippocampal plasticity referred to as long-term potentiation or LTP has provided an advanced understanding of the cellular and molecular underpinning of memory. Some of the early stages of memory loss preceding the later pathology of Alzheimer’s disease may involve inhibition of LTP, and insights about these molecular events may inform therapeutic approaches for cognitive enhancement neuroprotection.

## 1. INTRODUCTION

Despite the generally stereotyped and stable gross anatomy of the nervous system, one of its most intriguing features is its ability to change at the cellular level as a consequence of experience. The nature of this change has long fascinated neuroscientists. A particularly important kind of change involves information storage, a complex topic that, to streamline the discussion, we refer to simply as “memory.” There is compelling evidence that for short forms of memory, often referred to as working memory, information is stored by ongoing activity in neuronal networks (1–4). This process is used for moment-to-moment decision making and lasts for at most tens of seconds. The more enduring storage of information, however, remains intact after brain silencing. Thus, in cases where neuronal activity has been transiently silenced, as in barbiturate overdose (5) or brain cooling (6), memories remain intact. This finding suggests that the “memory” must be stored as a change at the cellular/molecular level, independent of activity. Theoretical models proposed that this could occur by a multimeric protein in which subunits phosphorylate one another (7, 8). However, it was not until the discovery of a Ca^2+^/calmodulin-dependent protein kinase, now referred to as CaMKII (9, 10), which became independent of Ca^2+^ (autonomous) after autophosphorylation (11–15) and exhibited switchlike autonomy after autophosphorylation of just a minority of its subunits (15), that these theoretical models gained a biological framework.

As the properties of CaMKII were being elucidated, long-term potentiation (LTP), in which brief repetitive synaptic stimulation results in long-lasting increases in synaptic strength, was gaining popularity as a cellular model for learning and memory (16–22). As explained more fully below, the prevailing hypothesis for synaptic strengthening is that there is an increase in the number of α-amino-3-hydroxy-5-methyl-4-isoxazolepropionic acid (AMPA)-type receptors (AMPARs) present at glutamatergic synapses. Here we review the fascinating story of how these two lines of research, LTP and CaMKII, evolved and finally merged. The overall goal is to *1*) integrate the remarkable biochemical properties of CaMKII with the equally compelling properties of LTP and *2*) highlight areas where critical gaps in our knowledge remain. The reader is referred to excellent reviews that emphasize the biochemistry and structure of CaMKII (23–30) and the properties of LTP (17–19, 22, 31, 32).

## 2. LONG-TERM POTENTIATION: THE BASICS

**Figure 1.**
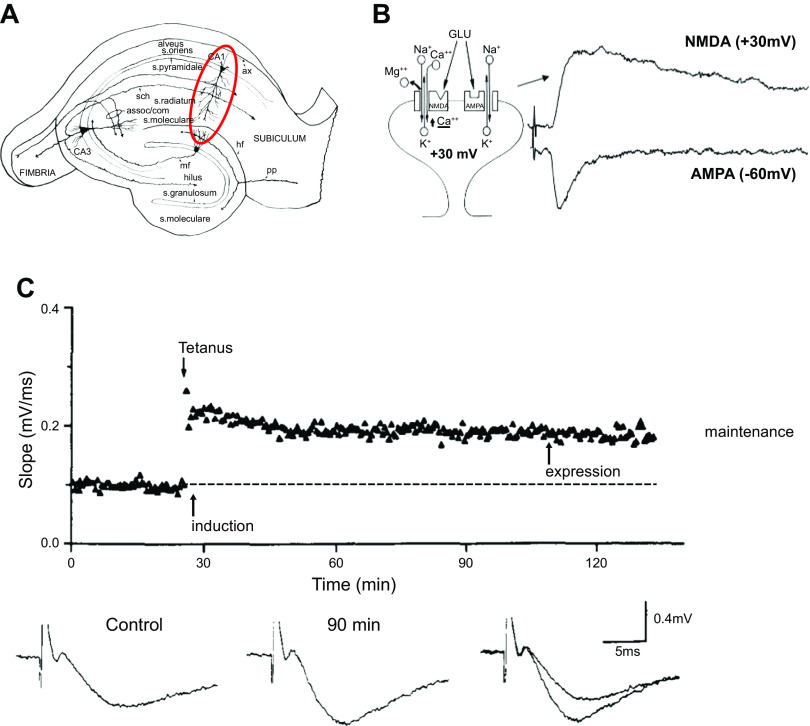
A diagram of the hippocampal slice and example of long-term potentiation (LTP). *A*: diagram of the hippocampal slice preparation. The red circle highlights the CA1 region where most studies on LTP have been carried out. *B*: diagram of an excitatory spine synapse onto a dendritic spine. Synapses contain α-amino-3-hydroxy-5-methyl-4-isoxazolepropionic acid (AMPAR) and *N*-methyl-d-aspartate (NMDAR) receptors. Glutamate (GLU) binds to both receptors, but at resting potentials (−60 mV) only the AMPAR is activated, because the NMDAR channel is blocked by extracellular Mg^2+^. Depolarizing the cell (+30 mV) unblocks the NMDAR. *C*: an example of LTP. Field potential responses (aggregate extracellular responses reflecting the simultaneous activation of a large number of excitatory synapses) are shown at *bottom*. After a 25-min baseline of responses evoked by 0.1-Hz stimulation, a 1-s, 100-Hz tetanus is delivered and then 0.1-Hz stimulation is resumed for the rest of the experiment. The response is measured as the slope rather than the peak to avoid voltage-dependent responses that can contaminate the measurement. The response to 0.1-Hz stimulation following the tetanus is clearly elevated, as shown by the superimposed traces on *right*. Modified from Ref. 38, with permission from *Neuron*.

Here we provide a brief review of the mechanisms underlying long-term potentiation (LTP). LTP, discovered half a century ago (33, 34), has many extraordinary properties that make it the most compelling cellular model for learning and memory currently known. Most importantly, it has the associative property, first predicted by Hebb (35) for a synaptic mnemonic mechanism. Originally discovered in the dentate gyrus of the hippocampus in anesthetized rabbits, it is now known to occur at excitatory glutamatergic synapses throughout the brain. LTP represents a marked increase in synaptic strength that follows a brief high-frequency train of electrical stimuli (a “tetanus”) delivered to excitatory fibers synapsing on postsynaptic neurons. Since then, for technical reasons, the mechanistic studies on LTP have focused mainly on the CA3-to-CA1 synapses onto hippocampal pyramidal cells and are limited for the first hour (see FIGURE 1). The introduction of the in vitro hippocampal slice (36) and the demonstration that LTP remained intact in the slice preparation (37) were essential in pursuing the cellular and molecular basis of LTP. The sequence of events in LTP is often divided into three phases: induction, expression, and maintenance (FIGURE 1) (38). Induction refers to the events that occur during the tetanus and addresses the way that the tetanus triggers the potentiation (learning). Expression addresses the question: In what way has the synapse changed to account for the potentiation? Maintenance addresses the question: What drives the synaptic strength to maintain the potentiation (the memory)?

### 2.1. LTP: Induction

LTP is pathway specific; neighboring inactive synapses fail to potentiate (19). Another property of LTP is cooperativity (39). If one tetanizes just a single or a few axons, the activated synapse(s) on a given postsynaptic cell fails to express LTP, no matter the frequency of stimulation. However, LTP is induced when the stimulus strength is increased so that many more axons are activated. The basis for this cooperativity was found to have a simple explanation: the stronger stimulation involves more axons and causes a much larger depolarization of the postsynaptic cell. Initially it was thought that the induction of LTP requires a tetanus of presynaptic axons. However, it is now known that LTP can be induced by just depolarizing the postsynaptic cell with current through the recording electrode combined with low-frequency synaptic stimulation (40, 41). Importantly, this same depolarization in the absence of synaptic stimulation fails to induce LTP. Thus, LTP requires that two events occur together: *1*) synaptic activation and *2*) postsynaptic depolarization. These properties are entirely consistent with a theoretical synaptic learning rule proposed by D. O. Hebb (35), in which the strength of a synapse can be enhanced by coactivation of pre- and postsynaptic partners. The mechanism underlying these features relies on the *N*-methyl-d-aspartate (NMDA) type of glutamate receptor (NMDAR), which is known to be required for LTP (42). Glutamatergic synapses primarily contain two types of glutamate receptors, AMPA receptors (AMPARs) and NMDARs (FIGURE 1*B*). Ordinarily excitatory drive is mediated primarily by AMPARs, even though glutamate binds to both types of receptors. The reason for this is that the NMDAR ion channel is blocked by the binding of Mg^2+^ to the external side of the receptor at normal cellular resting potentials. During the depolarization caused by a tetanus (or direct depolarization of the membrane by current injection through the recording electrode) the Mg^2+^ ion is electrostatically driven from its binding site, relieving the channel block. As discussed in sect. 3, NMDARs are highly permeable to Ca^2+^ and it is the rise in intracellular Ca^2+^ that provides the trigger for LTP. This accounts for the initial steps in LTP induction. The mechanism underlying induction and the essential role of the NMDAR were rapidly solved and universally agreed upon. Much of this review focuses on the events initiated by NMDAR activation.

### 2.2. LTP: Expression

We now turn to expression. In what way is synaptic transmission changed after LTP has been induced? This has been a contentious topic. The central question is whether the change occurs presynaptically, via an increase in glutamate release, or postsynaptically, via an increase in the sensitivity to glutamate. Many reviews have addressed this controversy (17–19, 22, 31). This controversy, which lasted well over a decade, was the first of two major roadblocks that impeded progress in understanding LTP. Although it is difficult to entirely exclude the possibility of a minor presynaptic component of NMDAR-dependent LTP, the evidence is overwhelming that its expression is primarily postsynaptic. Virtually all current research is focused on postsynaptic mechanisms. Although there is much discussion about the various important signaling pathways engaged by NMDAR activation, there is now broad consensus that the expression of LTP represents an increased accumulation of AMPARs at the potentiated synapse.

### 2.3. LTP: Maintenance

Finally, we turn to LTP maintenance. This is of fundamental importance, because it is the long-lasting nature of the potentiation that makes LTP such an attractive cellular model for information storage. Maintenance was the second of the two major roadblocks, lasting for over two decades, hampering our understanding of LTP. It is well accepted that CaMKII is required for the induction of NMDAR-dependent LTP, and the property of autonomy made CaMKII an ideal candidate for maintaining LTP. However, numerous attempts over the years to link CaMKII to the maintenance of LTP failed. Based on recent evidence, however, we argue that the CaMKII-NMDAR complex can indeed act to drive and maintain LTP (see sect. 9).

This review is limited to NMDAR-dependent LTP, because the Hebbian properties of this form are the most appealing in terms of synaptic memory storage. Additionally, NMDAR-dependent LTP has been most tightly associated with intact animal behavioral memory (19, 43–46). However, before turning to NMDAR-dependent LTP, it may be helpful to step back and view LTP in a broader perspective. There is admittedly considerable confusion that has plagued the field of LTP (47). This is primarily due to the existence of multiple forms of LTP, some more firmly established than others. It is well established that NMDAR-dependent and NMDAR-independent forms of LTP exist at different types of synapses [e.g., NMDAR-independent LTP at hippocampal mossy fiber synapses (48)]. There is even evidence that at CA1 excitatory synapses both NMDAR-dependent and NMDAR-independent forms of LTP can coexist under certain conditions (49–51). Perhaps the most common practice in the field is to divide LTP into “early” (the first hour) and “late” (after the first hour) forms. The mechanistic distinction between these forms has focused largely on the role of protein synthesis. It is proposed that LTP is independent of protein synthesis in the first hour but thereafter is dependent on protein synthesis (52–57). However, it has been argued that the distinction between early and late LTP is poorly characterized (58). Furthermore, although rarely cited, there are two rigorously controlled studies that failed to find any role for protein synthesis in late LTP (59, 60). Given this confusion, this review focuses solely on NMDAR-dependent LTP, and primarily on the first hour, since this has received the most attention.

## 3. CaMKII: STRUCTURE AND REGULATION

**Figure 2.**
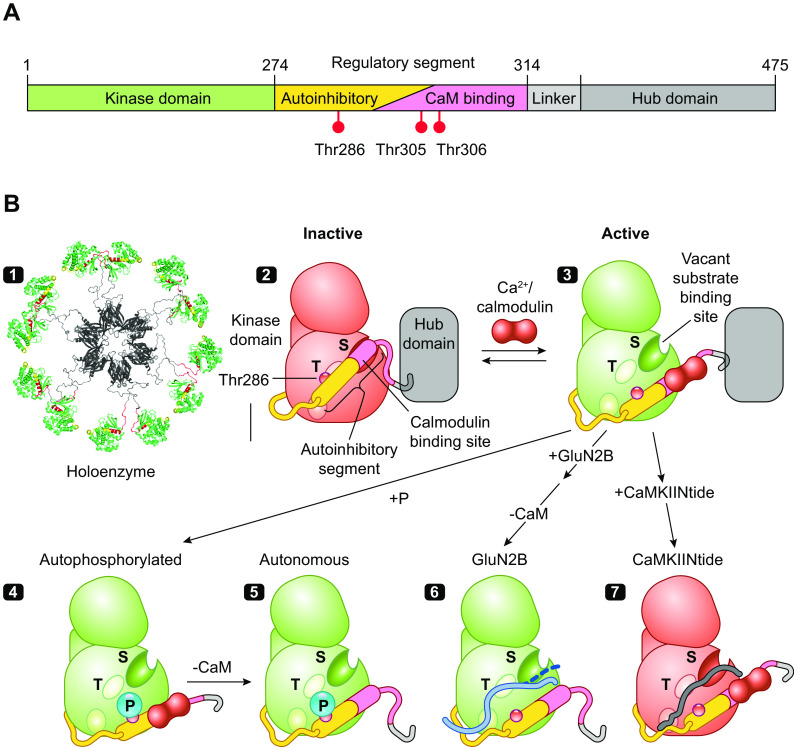
The structure and regulation of Ca^2+^/calmodulin (CaM)-dependent protein kinase IIα (CaMKIIα). *A*: the domain structure of CaMKII with kinase domain followed by regulatory segment, linker, and hub domain. *B*: schematic diagram showing the structural states of one CaMKII subunit (modified from Ref. 28, per terms of *Cold Spring Harbor Perspectives in Biology*) *1*: The dodecameric structure is formed by hub domains associated as 2 stacked hexameric rings (viewed from above the rings) with pairs of kinase domains from upper and lower ring radiating out via flexible linkers of variable size (PDB: 5U6Y). *2*: CaMKII is inactive in the basal state because of conformational and steric effects of its autoinhibitory segment. *3*: Ca^2+^/CaM binding activates CaMKII by competing with kinase domain for binding of the inhibitory regulatory segment, thereby activating the kinase and exposing a surface grove with 3 docking sites (shown as oval surface indentations), including the substrate binding pocket (S) and the general area where T286 was buried (T) that with the third site enables binding of GluN2B and some substrates and inhibitors. *4* and *5*: Active CaMKII can then be subject to several modifications or interactions, including autophosphorylation at T286 (*4*) and dissociation of Ca^2+^/CaM, leaving a T286P kinase that remains active (autonomous activity) (*5*). *6*: The activated state (autophosphorylated or not) can bind the COOH-terminal tail of GluN2B on a surface groove and T site previously occupied by the regulatory segment (61). GluN2B-bound kinase, measured after removal of Ca^2+^/CaM, remains active, suggesting only weak binding of COOH-terminal tail at the S site (dashed line). *7*: By contrast, CaMKIINtides bind across the entire groove, including the S site (62) to inhibit the kinase.

Discovered over 40 years ago (9, 10) as a multifunctional Ca^2+^/calmodulin-dependent protein kinase activity later termed CaMKII, it has some remarkable biochemical properties. First, although typical of CaMKs in having an NH_2_-terminal kinase domain followed by a CaM-binding regulatory domain, it is unique in having a COOH-terminal hub or association domain that assembles 12 subunits as a double-ringed, yoyolike holoenzyme (FIGURE 2, *A* AND *B*) (28). Second, it is present in the brain at extraordinarily high levels, rivaling the levels of cytoskeletal proteins (63–67), suggesting that CaMKII may play a structural role (27, 68). Third, stimulation of the kinase by brief spikes of Ca^2+^/CaM achieves activation that is dependent on spike frequency even when total exposure to Ca^2+^/CaM is kept constant (69). Fourth, CaMKII has the intriguing property that once activated by Ca^2+^ the enzymatic activity toward substrates remains after Ca^2+^ removal (11–15). This immediately suggested a role in information storage (15, 70).

As is the case with many kinases, CaMKII contains an autoinhibitory regulatory segment (FIGURE 2, *A*, *B1,* AND *B2*) that binds across the substrate binding groove, whose structure and function have been delineated by peptides, mutations, and crystallography (71–77). The autoinhibitory segment serves to strongly inhibit the kinase by locking it in a conformation that is not optimal for its phosphotransferase activity, reducing affinity for ATP, occluding substrates from the active site, and constraining binding of CaM to an incomplete binding site in the regulatory segment (FIGURE 2*B2*). The binding of Ca^2+^/CaM peels off the regulatory segment from the surface of the kinase, thus unlocking constraints on catalytic competency and providing access to substrates and CaMKII interacting and anchoring proteins (e.g., GluN2B, Tiam1, and CaMKIIN) (61). The structure of a CaMKIIN peptide to CaMKII shows it to bind across the surface groove, with docking sites designated as A, B, and C (3 oval indentations in FIGURE 2) (62). The CaMKII amino acid residue numbers here and elsewhere in the text refer to CaMKIIα. To simplify discussion of the surface groove and conform to prior designated landmarks, we refer to the core substrate recognition and phosphotransferase site as S (A site) and the residues previously sheltering T286 from phosphorylation as T (approximately B site) (FIGURE 2*B3*). Kinase activation changes the conformation of this region, but it is colloquially still often referred to as the T site, e.g., as part of a surface groove and pocket interacting with GluN2B (78) and some inhibitors we discuss below.

The action of displacing the autoinhibitory domain also exposes and helps to present T286 to a different active subunit that autophosphorylates it. This autophosphorylation is primarily intersubunit autophosphorylation within a holoenzyme, although at very high kinase concentration it can also occur by an interholoenzyme mechanism (FIGURE 2*B4*) (71, 79–81). Phosphorylated T286 disables the ability of the autoinhibitory segment to return to the autoinhibited state of the kinase after dissociation of Ca^2+^/CaM, so the kinase remains in an open or active configuration (FIGURE 2*B5*). Thus, although a Ca^2+^ signal is essential for activation of the kinase in the first place, activity of CaMKII with P-T286 is autonomous of Ca^2+^. The phosphorylation of T286 requires that the subunit being phosphorylated have Ca^2+^/CaM bound (82, 83), resulting in autophosphorylation. It is generally believed that Ca^2+^/CaM binding and peeling of the regulatory segment is necessary for exposure of T286. Importantly, as discussed below, under certain conditions the requirement for Ca^2+^/CaM binding for intersubunit phosphorylation may not be absolute.

Two additional autophosphorylation sites, T305 and T306, are largely spared during activation by Ca^2+^/CaM as these are within the CaM binding segment. However, once Ca^2+^/CaM dissociates from a kinase phosphorylated at T286, its autonomous activity quickly phosphorylates T305 and T306, blocking rebinding of Ca^2+^/CaM and exerting a brake on further Ca^2+^/CaM stimulation, in what is at times referred to as inhibitory autophosphorylation (80, 84–86). However, it is important to note that T305/T306 phosphorylation does not directly inhibit kinase activity (84, 86). The specific role for T305/T306 in LTP has not been established. However, T305A/T306A mutations increase CaMKII levels in PSDs and lower the threshold for LTP (87). During glutamate-induced translocation of CaMKII in culture, these same mutations greatly increase CaMKII resident time at synaptic sites (88).

Autophosphorylation of T286 markedly increases the affinity of the kinase for Ca^2+^/CaM. As indicated above, structural constraints prevent Ca^2+^/CaM from interacting with its full binding site in a naive CaMKII. After T286 autophosphorylation, however, the autoinhibitory segment may be further displaced from the kinase surface so that the full CaM binding site becomes available (74, 89, 90) and the affinity for Ca^2+^/CaM increases >1,000-fold (from 15 nM to 20 pM) (91). The increased affinity is due to a greatly reduced dissociation rate of Ca^2+^/CaM; it takes many seconds to dissociate when Ca^2+^ is elevated versus a fraction of a second before autophosphorylation and is referred to as CaM trapping (91, 92).

### 3.1. CaMKII and Integration of Ca^2+^ Stimuli

**Figure 3.**
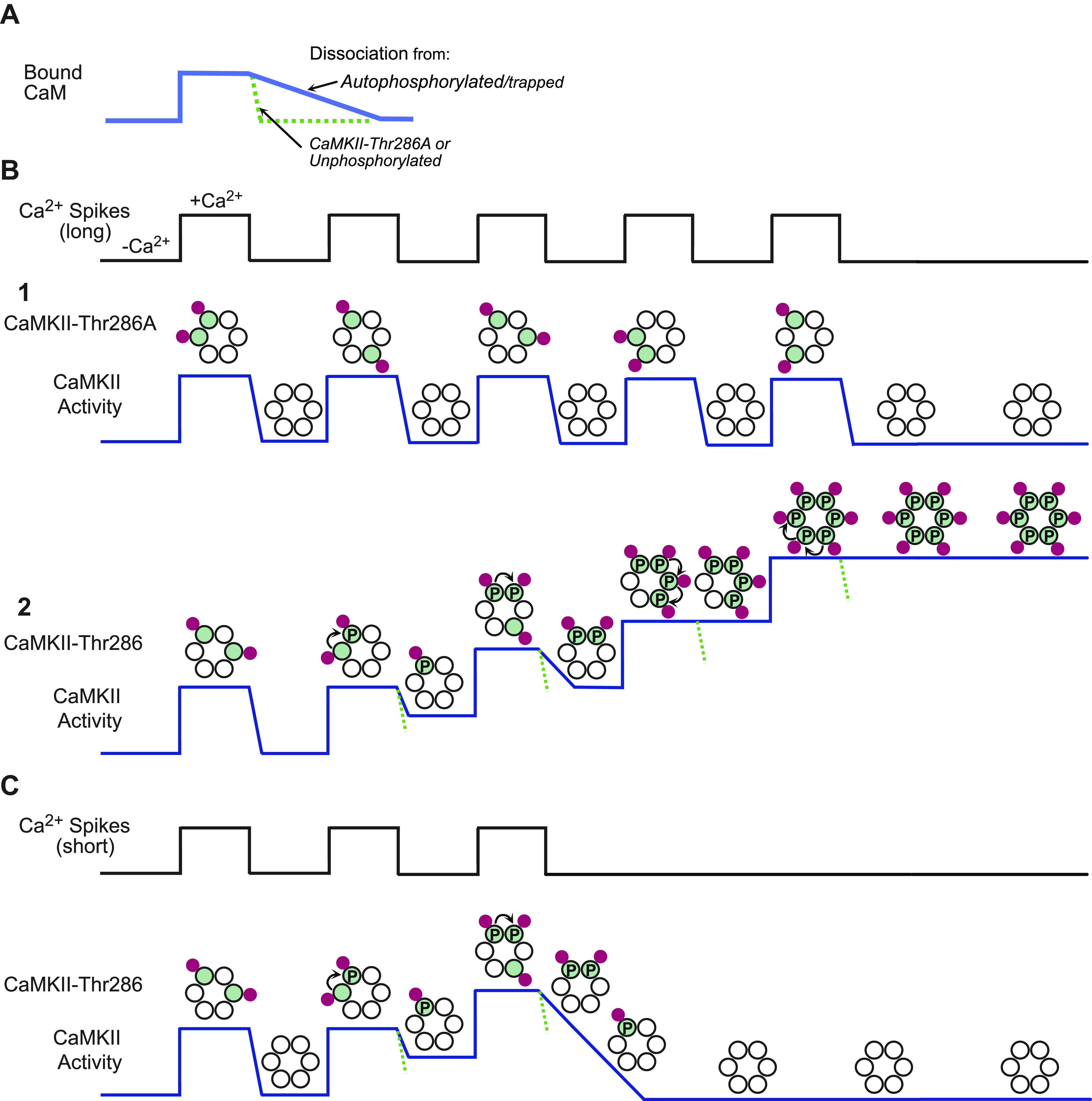
Ca^2+^/calmodulin (CaM)-dependent protein kinase II (CaMKII) and integration of Ca^2+^ stimuli. *A*: graph illustrating that CaM binds CaMKII in response to a rise in Ca^2+^ and quickly dissociates as the Ca^2+^ level returns to baseline if the kinase is a phosphonull (T286A) or an unphosphorylated wild-type subunit. By contrast, dissociation of CaM is greatly slowed by autophosphorylation, because it greatly increases CaM affinity primarily by reducing the dissociation rate, termed CaM trapping. *B*: an example of a train of high-frequency Ca^2+^ pulses used to illustrate the effect of autophosphorylation. In subpanels, red dots represent Ca^2+^/CaM; green dots are CaM bound (active) subunits; P represents autophosphorylated (active) subunits; and dashed green line indicates rate of kinase deactivation of unphosphorylated holoenzymes. Each Ca^2+^ pulse leads to binding of CaM to some of the subunits in a holoenzyme (shown as 1 hexameric ring for simplicity) and partially activating CaMKII. *B1*: with T286A the critical autophosphorylation does not occur and activity drops to baseline between each Ca^2+^ pulse so that each pulse averages the same level of activation without integration of stimuli. *B2*: trapping of CaM and integration of activity by rapid Ca^2+^ spikes. Intersubunit autophosphorylation only occurs when the subunit to be phosphorylated has a proximate neighbor that is simultaneously active. In this example, autophosphorylation does not occur in the first Ca^2+^ pulse but does so in the subsequent Ca^2+^ pulses. CaM trapping by an autophosphorylated subunit effectively increases the probability that when CaM binds at the next Ca^2+^ pulse it will be on a subunit proximate to an active kinase subunit, further increasing autophosphorylation and CaM trapping ahead of the next Ca^2+^ pulse. Thus, beyond a threshold frequency, successive Ca^2+^ pulses will recruit more CaM to CaMKII to produce a highly active (and autonomous) kinase that remains active in the absence of further Ca^2+^ pulses (autonomy). *C*: the same as in *B2*, but the Ca^2+^ pulses stop when CaMKII is partially activated. In this case the partially active enzyme is vulnerable to phosphatases and is dephosphorylated. Modified from Ref. 93, with permission from *Science*.

A prediction of the basic properties of CaMKII described above is that with brief repetitive Ca^2+^ spikes the enzyme would recruit more Ca^2+^/CaM, increasing the probability of autophosphorylation and a higher level of activity (FIGURE 3). By comparison, long intervals between Ca^2+^ spikes, which allow dissociation of Ca^2+^/CaM or a phosphonull mutant, e.g., T286A, would not exhibit increasing activity with a train of repetitive Ca^2+^ spikes. In essence, T286 autophosphorylation and CaM trapping would produce a frequency dependence for CaMKII activation. This has, in fact, been demonstrated with purified CaMKII subjected to different frequencies and durations of Ca^2+^ spikes (69, 94). CaMKII isoforms exhibit different frequency dependencies resulting from different linker lengths that determine how extended the catalytic domains are from the hub and each other. This frequency dependence would potentially enable CaMKII to decode frequency of synaptic inputs.

The basis for the demonstrated frequency dependence has not been rigorously established, but a scenario showing how CaM trapping could support frequency dependence is illustrated in FIGURE 3. It shows levels of CaMKII activity at a Ca^2+^ spike frequency that allows integration of activity during a spike train. It is based on a rapid dissociation of Ca^2+^/CaM from an unphosphorylated subunit (or T286A mutant) and a greatly reduced dissociation if some of the subunits are autophosphorylated. A reasonable assumption is that during a brief Ca^2+^ spike there is only time for a fraction of a dodecamer to be occupied and activated by Ca^2+^/CaM. Activation is also curtailed by insufficient cellular free CaM for all its binding proteins, since a significant fraction of CaM is membrane bound and not available in the cytosol, and the relatively low affinity of unphosphorylated CaMKII for the free Ca^2+^/CaM (95).

We first look at T286A (FIGURE 3*B1*), in which autophosphorylation that traps CaM cannot occur. It shows a sequence with increased Ca^2+^ corresponding to the increasing number of activated subunits and rapidly deactivates to baseline after Ca^2+^ levels decline. Each Ca^2+^ spike produces the same degree of activation without summation of activity with successive spikes. By contrast (FIGURE 3*B2*), with each spike there is a chance of autophosphorylation on CaMKII-T286, e.g., when one subunit is active while a neighboring subunit also has Ca^2+^/CaM. Overall, there is recruitment of CaM and higher activation can be achieved because autophosphorylated subunits trap CaM for some time. Hence successive spikes increase the total number of active or CaM-trapping subunits, thereby increasing the probability of autophosphorylation, CaM trapping, and total number of active subunits. At the end of a series of spikes T286A shows the same level of activity as after the initial spike, whereas CaMKII wild type (wt) would be expected to integrate stimuli and achieve increased total activity with each spike. This representation is strikingly similar to actual CaMKII responses in synaptic spines stimulated by repetitive glutamate uncaging and, as in FIGURE 3, shows little integrated activation with T286A CaMKII, unless substantially higher frequencies are used (94). The comparisons with T286A make it clear that autophosphorylation of T286 enables both a fuller activation of CaMKII during a stimulus train and a persistence of that activity even after the Ca^2+^ level returns to baseline.

Thus, when comparing the action of wt versus T286A, one should consider not only a fundamental difference in autonomous activity, but also a difference in the number of subunits being activated in response to repetitive stimulation. It is proposed that when only a few subunits in a holoenzyme get phosphorylated, autophosphorylation fails to exceed the rate of dephosphorylation and CaMKII activity decays back to baseline (FIGURE 3*C*) (64). Surprisingly, there have been few physiological studies addressing the possible integrative properties of CaMKII and its possible role in synaptic transmission. A very likely role is in metaplasticity (96–98). For instance, weak repetitive synaptic stimulation can elicit an NMDA-dependent decaying potentiation referred to as short-term potentiation (STP) (99–101). In addition, prior synaptic stimulation, which is not sufficient to change synaptic strength, can decrease the threshold for subsequent LTP (102). Such findings might well help explain a recently described form of plasticity referred to as behavioral timescale synaptic plasticity (BTSP) (103–106), which is responsible for the rapid formation of hippocampal place fields. It is NMDAR dependent, but unlike classical LTP the coincidence time window is seconds, not milliseconds. The possible role of CaMKII in this long time window is discussed further in sect. 11 on hippocampal place fields. It is assumed that with the near-saturating pairing protocol used to induce LTP the majority of the subunits of a holoenzyme would be phosphorylated (see last Ca^2+^ pulse in FIGURE 3*B2*).

### 3.2. Ca^2+^ Sensitivity of CaMKII

In considering the role of CaMKII in LTP, it is of paramount importance that the Ca^2+^ signal that initiates the potentiation come solely from the NMDAR. If potentiation were to occur from non-NMDAR sources, synapse specificity and the Hebbian nature of LTP would be lost. Thus, it is essential to compare the sensitivity of CaMKII to Ca^2+^ relative to the physiological changes in Ca^2+^ levels that occur in a dendritic spine. The major conclusion is that CaMKII has low intrinsic activity in the basal state and only achieves significant activity following induction of LTP and the NMDA-mediated Ca^2+^ influx. Basal CaMKII activity is also suppressed because the level of free CaM is far lower than that of total CaM. The high level of CaMKII and other CaM-binding proteins at the synapse also means that CaM is limiting. Much of CaM in spines is sequestered by proteins such as neuromodulin (GAP-43) and neurogranin. This and other aspects of the regulation of CaM and CaMKII have been nicely reviewed (107). Despite the fact that purified CaMKII preps have some minimal level of autophosphorylation, constitutive activity is typically <1% of total stimulated activity, i.e., >100-fold stimulation by addition of Ca^2+^/CaM. The Ca^2+^ sensitivity of CaMKII is well suited for its role in LTP. The low affinity of CaMKII for Ca^2+^ when CaM is not limiting (apparent *K*_d_ = 1.6 µM for autophosphorylation) (108) relative to the resting level of intracellular Ca^2+^ (20–50 nM) (109–111) and the unusually high Hill coefficient of ∼6 for CaMKII (108) ensure maintenance of the synapse specificity and Hebbian nature of LTP (19). This is despite the fact that CaM can activate CaMKII when only two of its four Ca^2+^ binding sites are occupied (112, 113).

The affinity for Ca^2+^/CaM is (at saturating Ca^2+^) far lower than the average Ca^2+^/CaM-dependent enzyme, e.g., *K*_d_ for CaM interaction with calcineurin = 28 pM; myosin light chain kinase (MLCK) = 5.1 nM; CaMKII = 51 nM; and CaMKII (T286P) = 7.6 pM (108, 114). This would suggest that significant CaMKII activation does not occur in quiescent cells. As indicated above, CaMKII has a relatively low affinity for CaM due to steric interference with CaM interactions until the kinase is autophosphorylated and its CaM binding site is fully exposed, leading to very high affinity and CaM trapping. CaM is less abundant than the sum total of its interacting proteins, so there is, in essence, a competition for CaM. A highly abundant protein, neuromodulin, functionally limits free CaM because it is highly abundant and sequesters CaM at basal Ca^2+^ only to release it as Ca^2+^ increases. It has been suggested based on modeling that neurogranin, another CaM “buffering” protein, ultimately facilitates CaMKII activation by localizing CaM, albeit initially bound, at the spine so that its availability does not require translocation of CaM from the shaft (115).

CaMKII is not competitive for the limiting Ca^2+^/CaM in its basal state but markedly increases its affinity as it becomes autophosphorylated. Given the high concentration of CaMKII at the synapse, one can anticipate that one effect of autophosphorylation, with the consequent increase in CaM affinity, is that CaMKII may suppress activation of other CaM-dependent enzyme by competing for Ca^2+^/CaM. Finally, the capacity of neuromodulin to “buffer” CaM is regulated by PKC, which can phosphorylate the CaM target site on neuromodulin (IQ domain) to reduce its CaM affinity and increase free CaM. In some systems it has been shown that pathways that stimulate PKC and release bound CaM can potentiate CaMKII (116, 117). Although the general outline of CaM integration of Ca^2+^ signals is known, there is a gap in the appreciation of the overall kinetics and dynamics of CaM-binding proteins at the synapse at various stages of LTP, including: the competition for CaM, formation of protein complexes, including via liquid-liquid phase separation (LLPS), and how processes such as LTP can be modulated by signaling pathways that regulate a Ca^2+^/CaM homeostasis.

### 3.3. Impact of NMDAR Binding to CaMKII

A second mechanism proposed for generating autonomy involves binding of the GluN2B subunit of the NMDAR to the kinase surface groove in place of the autoinhibitory domain that allows for autonomous activity without autophosphorylation (78). The initial finding used a stepwise procedure of first docking CaMKII to GluN2B COOH-terminal domain (CTD) peptides in the presence of Ca^2+^/CaM but without ATP. Ca^2+^/CaM was then removed and when the CaMKII-CTD complex was assayed with substrate and ATP it was found to be autonomously active (78). A similar finding was made with T286A, the phosphonull mutant, i.e., the CTD peptide induced constitutive activity without T286P. One explanation is that displacement of the regulatory segment by Ca^2+^/CaM enables the CTD peptide, which has homology to the regulatory segment, to bind in its place. The CTD peptide may act like a wedge between the kinase and its regulatory segment, propping the enzyme in an open active conformation that persists as long as the CTD peptide is bound, even after Ca^2+^/CaM dissociates (FIGURE 2*B6*) (78).

This finding is paradoxical given the crystal structure of CaMKII-CTD (61). As proposed, the CTD peptide does bind in place of the regulatory domain, across the entire groove on the kinase, including the active site (61). In other words, the GluN2B CTD should inhibit the kinase rather than make it constitutively active. Indeed, induction of autonomy following stepwise docking was replicated in an independent study, but it found, however, that simply adding CTD to a kinase reaction produced an inhibition of substrate phosphorylation (118). Inhibition is exactly what one finds with a peptide derived from an endogenous inhibitory protein termed CaMKIIN that binds like the CTD (FIGURE 2*B7*) (see sect. 3.5). For the CTD peptide to directly produce a constitutive kinase, one needs to propose that one end of the GluN2B CTD may function as a wedge at the T site to block reassociation of the autoinhibitory segment while its other end is not tightly bound to the active S site so that substrates can bind. This weaker interaction with the S site is illustrated as a dashed line on the GluN2B CTD in FIGURE 2*B6*.

As described above and discussed below (see sect. 9.2.1), although binding to GluN2B can be achieved without the phosphorylation of T286, under normal conditions T286 is expected to be phosphorylated after activation, translocation, and binding GluN2B. However, the state of T286 phosphorylation on GluN2B-bound kinase following the return of Ca^2+^/CaM to normal levels has not been fully resolved. Specifically, does T286 need to be phosphorylated to maintain the stability of the CaMKII-GluN2B complex (see sect. 9.2.1)? The binding of CaMKII to the GluN2B serves another critical role by targeting CaMKII to the core of the postsynaptic density (PSD), a membrane thickening juxtaposed to the presynaptic active zone. This binding of CaMKII to GluN2B is critical for the synaptic enhancement caused by CaMKII (see sect. 6) (119–122). The interaction is therefore critical both for maintaining the active state as well as for localizing CaMKII to the PSD, and therefore for LTP. This further highlights the central role of the NMDAR in plasticity: not only as a trigger of the plasticity via its Ca^2+^ permeability but as a scaffold that is required to sustain the plasticity.

The state of activity of CaMKII subunits docked to NMDA receptors may only be determined if this can be measured in the context of the PSD. It is also important to understand whether the proposed constitutive enzymatic activity of such subunits leading to T286 phosphorylation is critical for maintaining the CaMKII-NMDAR complex. What is clear is that given the ∼40-fold higher concentration of CaMKII subunits than GluN2B subunits (see sects. 9.2.2 and 13.2), only a small proportion of potential autonomous activity at the synapse can be due to the direct effect of GluN2B on CaMKII activity. There are also steric constraints that would make it very difficult for GluN2B in the plane of the membrane to interact with all subunits of a holoenzyme whose subunits are arrayed in two rings, with catalytic subunits potentially in opposite orientations. What is likely more important is the formation of the CaMKII-NMDAR complex per se and its maintenance despite phosphatase activity and protein turnover, which we discuss below (see sects. 9.2.2 and 10).

In addition to forming a complex with GluN2B, CaMKII phosphorylates S1303 (123) in the CTD. Disrupting CaMKII binding prevents phosphorylation (124). The predominant interaction of CaMKII to GluN2B is not via the catalytic site (S site), as it is not blocked by syntide-2 (125); however, phosphorylation at S1303 does promote slow dissociation of CaMKII (123, 125, 126). Phosphorylation of S1303 in heterologous cells enhances NMDAR current (127, 128) and reduces desensitization of GluN2B-containing receptors (129). These effects in heterologous cells contrast with the finding that overexpression of constitutively active CaMKII has little effect on synaptic NMDAR responses (130–132). Furthermore, the finding that the binding of CaMKII to GluN2B is essential for its synaptic action (see sect. 6) and constitutive activity of CaMKII-GluN2B toward substrates suggests that either S1303 is not phosphorylated in the GluN2B-CaMKII complex or phospho-S1303 does not significantly prevent binding or inhibit activity. In summary, the role of S1303 phosphorylation remains enigmatic.

### 3.4. Self-Association of CaMKII

There is a long trail of studies showing that under certain conditions CaMKII can assemble into large clusters both in vitro and in vivo. Much of the in vitro work has focused on conditions such as ischemia, low pH, low ATP, etc. However, considerable evidence suggests that this phenomenon is not simply aggregation and may be important physiologically. Self-association is initiated by Ca^2+^/CaM (133) and is reversible (134). It occurs with CaMKIIα but not with CaMKIIβ (133). Although self-association is favored by low pH, it is strongly dependent upon kinase concentration (133), suggesting that in dendritic spines it could occur at normal pH. 

The requirements for clustering are consistent with the subunit capture conformation seen in a crystal structure of CaMKII truncated after a portion of the regulatory segment and lacking the CaM binding domain and the remaining COOH-terminal end of the protein (62). The monomeric units form a repeating chain of regulatory segment of one bound to the kinase groove of a second monomer and regulatory segment of that second monomer bound across the kinase groove of a third monomer, and so on. Similar interactions appear to occur in holoenzyme clusters but with the interacting pair being a kinase groove in a subunit of one holoenzyme and regulatory segment of another subunit in another holoenzyme, and so on, forming a cluster or network. Consistent with such a model for holoenzymes (134), clustering is inhibited by regulatory segment-derived peptides, e.g., AC-3 and AIP that bind at the T site, but is not inhibited by even high levels of syntide-2, which only binds at the catalytic or S site (133). Self-association occurring in transfected cells was used to show that the I205K mutant that blocks docking of GluN2B to the kinase also blocks clustering, suggesting that the regulatory segment and GluN2B have an overlapping docking site (134).

Two aspects of kinase structure reduce clustering, one is the CaMKIIβ subunit and another is T286 phosphorylation, but these are countered by the high CaMKII concentration at the PSD (134, 135). Although autophosphorylation has a negative effect on clustering in vitro (135), CaMKII in cellular clusters does contain T286P (134), suggesting that autophosphorylation does not prevent cluster formation. Self-association also occurs in neurons. Glutamate treatment of neuronal cultures causes CaMKII clustering at excitatory synapses (136–140). This clustering is reversible, but in the presence of phosphatase inhibitors it is longer lasting (141). Recent studies show that at high concentrations holoenzymes can interact and initiate interholoenyzme phosphorylation (81). This may be an enzymatic consequence of the cluster formation. A computational study provides evidence that self-association could provide a concentration-dependent switch to amplify CaMKII sequestration in the PSD (142).

The widely made observation that CaMKII translocates to PSD at the synapse, coupled with the finding that CaMKII binds the NMDAR, might suggest that translocation is accounted for by such direct holoenzyme-NMDAR interactions. Similarly, the finding that the NMDAR produces autonomous activity without autophosphorylation might suggest that much of the constitutive activity at the PSD following translocation is due to the NMDAR and not T286P. These perspectives warrant a consideration of the stoichiometry of CaMKII-GluN2B binding. It is assumed that with the standard saturating induction protocol used for LTP the kinase would be highly phosphorylated, perhaps only limited by the availability of CaM. However, since there are roughly 20–30 NMDARs in the PSD (66, 143) and ∼100 holoenzymes [∼1,200 CaMKII subunits (65)], after NMDAR activation only a small fraction of subunits per holoenzyme would be directly bound to GluN2B. Given the stoichiometry, most holoenzymes translocating to the PSD cannot be directly bound to NMDA receptors. But an NMDA-CaMKII interaction involving one holoenzyme can amplify the redistribution of more CaMKII to the synapse by self-association that engages holoenzymes not bound to NMDAR. We return to this issue in sect. 6.

### 3.5. CaMKII Peptide Inhibitors

To study the physiological role of CaMKII, two classes of inhibitory peptides have been engineered. The first class of peptides are modified autophosphorylation site sequences lacking a phosphorylatable residue referred to as autocamtide-2-related inhibitory peptides (AC2-I, AIP, AC3-I) (78, 144–148). The second class of peptides is derived from an endogenous protein that inhibits CaMKII with nanomolar potencies (CaMKIIN) (149). They were discovered by yeast two-hybrid screens as proteins interacting with CaMKII lacking the regulatory domain. The two isoforms of CaMKIIN have overlapping regional distribution with the two brain CaMKII isoforms. Inhibitory segments of CaMKIIN that retain CaMKII selectivity are useful diagnostic tools, although there is no direct evidence for their regulation of CaMKII function in vivo. These shorter fragments are referred to as CaMKIINtides and include CN27 and CN21 (145, 149–152) and are typically used with added cell internalization sequences. They inhibit kinase activity with high affinity by binding across the entire substrate binding site (FIGURE 2*B7*) (61, 147, 153), and therefore also block GluN2B binding (152, 154). The degree to which CaMKIINtides, GluN2B, and AIP use separate interactions to bind across the kinase domain remains unresolved (61).

### 3.6. Comparison of CaMKIIα and CaMKIIβ

There are four CaMKII isoforms, but the α- and β-isoforms are the most abundant isoforms in brain. In the forebrain the ratio is roughly 3 α to 1 β, and holoenyzmes are composed of both subunits (155–157). The main structural difference between these isoforms is that the α has a short hub linker whereas the β has a long hub linker. The linker length affects CaM affinity and Ca^2+^ spike frequency sensitivity (69, 158) as well as rates of autophosphorylation (159). Finally, CaMKIIα but not CaMKIIβ supports a structural function of CaMKII in translocation of proteasomes to synaptic sites (160) and clustering of CaMKII holoenzymes in response to low pH (133).

There are two other differences between CaMKIIα and CaMKIIβ. Inactive CaMKIIα, but not β, binds to Shank3, and when it is activated it dissociates (161). Shank3 resides in the pallium of the PSD, just below the core (139, 162). It is proposed that this CaMKIIα-Shank3 complex may serve as a nearby tethered reservoir pool of CaMKII (161). Interestingly, CaMKIIα can also bind to a distinct domain of Shank3, which plays a role in gene transcription (163). Deletion of Shank3 has been reported to inhibit LTP (164–166). A second difference is the specific high-affinity binding of the inactive β, but not α, to F-actin (167–170). Upon activation CaMKIIβ dissociates from F-actin because of competition with Ca^2+^/CaM for the F-actin binding region, and dissociation is made more persistent by specific autophosphorylation of CaMKIIβ in the F-actin binding region (171–174). Again, F-actin binding is thought to provide a reservoir pool of CaMKII holoenzyme. Interestingly, deletion of CaMKIIβ impairs LTP (121, 172, 175), as does the deletion of CaMKIIα. The defect caused by CaMKIIβ deletion cannot be rescued by a mutant form of CaMKIIβ that cannot bind to F-actin (172). Importantly, expression of CaMKIIβ in cells lacking both CaMKIIα and CaMKIIβ fails to rescue LTP (121), and yet expression of CaMKIIα in cells lacking both isoforms fully rescues LTP (121). Assuming that native CaMKII holoenzymes are composed of a mixture of α- and β-isoforms, how can we put together a coherent model for CaMKIIβ? Perhaps the β-isoform sequesters inactive CaMKII on F-actin and only when this trapped pool is released during activation can the action of CaMKII be expressed. Pure α is fully functional because it is not sequestered by F-actin. However, the failure of the pure β-isoform to rescue function indicates that the β-isoform lacks some critical component that is present in the α-isoform.

Why is CaMKIIβ unable to support LTP, given that it, like CaMKIIα, undergoes autophosphorylation resulting in Ca^2+^-independent autonomy? A recent study has addressed this question (122). Given that binding of CaMKII to GluN2B is critical for its action, perhaps CaMKIIβ is unable to bind to GluN2B. However, both isothermal titration calorimetry and glutathione S-transferase (GST) pulldowns indicate that the binding of both isoforms is the same. Remarkably, despite the equal binding, CaMKIIβ, in contrast to CaMKIIα, failed to undergo liquid-liquid phase separation when combined with GluN2B CTD, Ca^2+^, and CaM. The failure of CaMKIIβ to phase separate could be rescued by swapping its long hub linker with the shorter linker from CaMKIIα, and this chimera fully rescued LTP. These findings are intriguing because they show that the binding of CaMKII to GluN2B is not enough to form a stable functional complex that phase separates. The added requirement may be that holoenzymes bound to GluN2B also need to bind each other and form clusters, as this is a property of CaMKIIα but not of CaMKIIβ (133). CaMKIIα, with a smaller radius (from hub to catalytic domains) may enable better packing into clusters than the larger CaMKIIβ. This also suggests a correlation between structural features necessary for phase separation and for LTP, consistent with a role of phase separation in LTP. Although these results provide an explanation for why CaMKIIβ is unable to support LTP, we are still left with the conundrum as to what the synaptic role of CaMKIIβ is.

## 4. Ca^2+^ AND LTP

In much of this review the terms “necessary” and “sufficient” are used. In general, this means that a necessary condition is one that must be present in order for another condition to occur, whereas a sufficient condition is one that produces the said condition. This has a long usage in scientific research. However, we are aware that there has been much debate concerning the logistical rigor of its usage (https://plato.stanford.edu/entries/necessary-sufficient/). Despite these reservations, we feel that using “necessary” and “sufficient” is of heuristic value in our presentation.

### 4.1. Postsynaptic Ca^2+^ and Necessity

**Figure 4.**
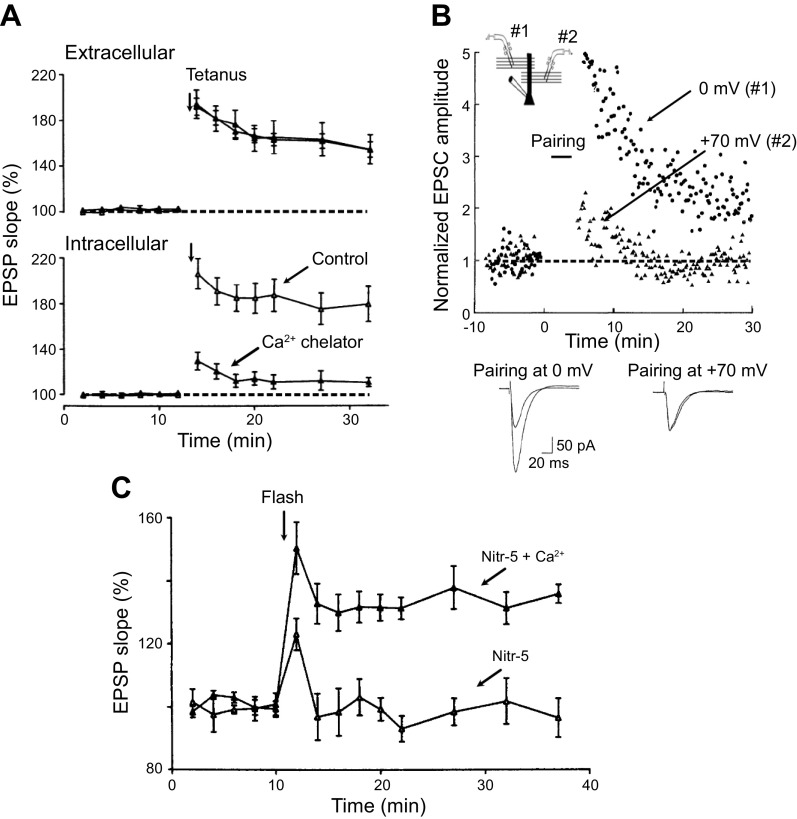
Ca^2+^ is necessary and sufficient for long-term potentiation (LTP). *A*: in this series of experiments an extracellular electrode records the field potential LTP from a population of neurons (*top*). Simultaneously, an intracellular electrode monitors LTP from a single neuron (*bottom*). When the intracellular recording electrode is filled with the standard electrolyte, the magnitude of LTP is the same as that recorded with the field electrode. In contrast, when the intracellular electrode contains nitr-5, a nitrobenzhydrol tetracarboxylate (a Ca^2+^ chelator), LTP is absent (*bottom*) (177), indicating that Ca^2+^ is necessary for LTP. *B*: the experimental design involves stimulating 2 independent pathways and recording responses from a single cell (see *inset*). The stimuli to the 2 pathways were alternated to get a baseline for each pathway. The cell is depolarized to 0 mV and stimulation of *pathway 1* continues, but stimulation of *pathway 2* is stopped. The cell is then depolarized to +70 mV and stimulation to *pathway 1* stopped, but stimulation of *pathway 2* commences. After this pairing protocol, the cell is returned to −70 mV. Robust LTP is observed for *pathway 1* but no LTP for *pathway 2*. Superimposed sample records of before and after pairing are shown below. The results indicate that a rise in Ca^2+^ through *N*-methyl-d-aspartate receptors (NMDARs) is instructive for LTP (178). *C*: a rise in postsynaptic Ca^2+^ is sufficient to potentiate synaptic transmission, Photo uncaging of Ca^2+^ from nitr-5, a nitrobenzhydrol tetracarboxylate Ca^2+^ chelator, enhances synaptic transmission (filled triangles) but fails to enhance synaptic transmission when the cage is not loaded with Ca^2+^ (open triangles) (177). EPSC, excitatory postsynaptic current; EPSP, excitatory postsynaptic potential. Images modified from Refs. 177 and 178, with permission from *Science* and *Neuron*, respectively.

The original experiment linking Ca^2+^ to LTP showed that loading postsynaptic cells with the Ca^2+^ chelator EGTA blocked LTP (176), suggesting that Ca^2+^ is necessary for LTP. Later experiments elaborated on this finding (177) (FIGURE 4*A*). In this experiment LTP was monitored both with an extracellular field potential recording, which records responses from a large population of neurons FIGURE 4*A*, *top*), and with an intracellular recording electrode to record responses in an individual neuron (FIGURE 4*A*, *bottom*). In the control experiment, the intracellular electrode contained the standard 3 M CsCl and the levels of LTP recorded with both recording electrodes are of similar magnitude.

However, when the intracellular electrode contained a Ca^2+^ chelator no LTP occurred in the individual cell, while normal LTP was recorded from the field electrode, which measures the response from a large population of cells. These findings indicate that postsynaptic Ca^2+^ is required for LTP, but they do not identify the source of the Ca^2+^. For instance, LTP might require a resting level of Ca^2+^ that is permissive for LTP, or it might require an actual elevation in Ca^2+^ above resting levels. The discovery that NMDARs are highly permeable to Ca^2+^ (179, 180) favors the latter but does not exclude the former. If an elevation of Ca^2+^ from an external source acts as an instructive signal, one would predict that strong depolarization of the postsynaptic cell toward the equilibrium potential for Ca^2+^, thus decreasing the driving force for Ca^2+^ and the decreasing Ca^2+^ influx, should reduce or prevent LTP. Such an experiment is shown in FIGURE 4*B* (178). In these experiments LTP was induced by the “pairing protocol” (40) rather than external tetanic stimulation, as this protocol allows for greater control over the depolarization that is required for LTP. Here the responses were produced by alternating low-frequency stimulation of two independent pathways that both impinge on the same neuron (FIGURE 4*B*, *inset*). In the first part of the experiment stimulation in *pathway 2* was stopped while stimulation continued in *pathway 1*. The cell was then depolarized to 0 mV, which is sufficient to remove the Mg^2+^ block from the channel while preserving a significant driving force for Ca^2+^. Hence 0 mV should allow near-maximal Ca^2+^ entry through the NMDAR. At this point stimulation was resumed in *pathway 2* and stopped in *pathway 1* and the cell was further depolarized to +70 mV. This strong depolarization will open the NMDAR channels while the reduction in Ca^2+^ driving force will effectively abolish Ca^2+^ influx. After the depolarization, robust LTP was recorded in *pathway 1* (0 mV), but no LTP was recorded in *pathway 2* (+70 mV). These experiments demonstrate that Ca^2+^ entry through the NMDAR is indeed not only necessary but instructive for LTP.

### 4.2. Postsynaptic Ca^2+^ and Sufficiency

The question of whether a rise in postsynaptic Ca^2+^ is sufficient to account for the ability of NMDARs to initiate LTP has a complex history. Three approaches have been used to address this issue. First, a photosensitive caged Ca^2+^ was employed (177, 181–183). Photostimulation of cells loaded with the caged Ca^2+^ compound nitr-5 without depolarization of the cells enhanced responses (FIGURE 4*C*) (177). Further studies showed that nitr-5 can evoke either an enhancement or a depression, depending on the amplitude and the duration of the Ca^2+^ signal (181). Brief large-amplitude signals favor potentiation, whereas more modest long-duration signals favor depression (181). Furthermore, it was found that prior LTP occluded the photo-induced potentiation (182). In the second approach, cells were loaded with Ca^2^/CaM from the recording electrode (184). This caused a slowly developing enhancement that occluded LTP. In addition, synapses expressing LTP are not enhanced by Ca^2+^/calmodulin. These first two approaches strongly support the proposal that a rise in postsynaptic Ca^2+^ is sufficient for LTP.

The third approach involved repeatedly activating voltage-dependent Ca^2+^ channels (VDCCs) with depolarizing pulses in the presence of NMDAR antagonists to raise spine Ca^2+^ levels. Activating VDCCs, which are present in spines, evokes cytosolic spine Ca^2+^ transients (185–187) and the activation of cytosolic spine CaMKII (188). Although voltage pulses clearly potentiate excitatory postsynaptic currents (EPSCs), referred to as voltage pulse potentiation (VPP), the potentiation typically is transient, lasting no longer than 20 min (Refs. 189–193, but see Refs. 194, 195). Furthermore, VPP does not occlude with LTP (189, 192), strongly implying a mechanism distinct from LTP. Interestingly, VPP requires CaMKII. Most significantly perhaps, the addition of phosphatase inhibitors makes the potentiation long lasting (191). Although it is difficult to provide a perfectly satisfactory model to explain these results, one plausible hypothesis is that there are two nonoverlapping Ca^2+^ nanodomains: one at the mouth of the NMDAR and the other at the mouth of the VDCC. The signaling in the latter is less sensitive to Ca^2+^, such that nitr-5-induced Ca^2+^ transients would fail to activate it. Furthermore, phosphatases would prevent any cross talk between the two domains. The local CaMKII at VDCCs would activate a signaling pathway that, although involving CaMKII, is independent of LTP, and can enhance AMPAR responses. The addition of phosphatase inhibitors allows the VDCC-activated CaMKII to spread to the NMDAR nanodomain and engage LTP. If such a hypothesis is correct, then phosphatase inhibitors would give rise to long-lasting VPP, which would be occluded by LTP. The early potentiation would not be occluded by LTP. These predictions are consistent with observed effects. Such a hypothesis could also explain the finding that with intense induction protocols for LTP a component of LTP requires the activation of VDCCs (49–51, 193, 196). An important task for the future will be to test this hypothesis directly.

## 5. CaMKII AND THE INDUCTION OF LTP

### 5.1. CaMKII and Necessity

If Ca^2+^ is necessary and sufficient to account for the ability of NMDARs to initiate the process of LTP, what is/are the downstream target(s) of Ca^2+^? Several Ca^2+^-dependent protein kinases are expressed in hippocampal neurons [e.g., PKA (via adenylyl cyclase subtypes 1 and 8), PKC, CaMKII]. The high levels of CaMKII and its unique biochemical properties focused attention on this particular kinase. Both pharmacological and genetic approaches have been used to address its role in the induction of LTP. Pharmacological studies employed two series of potent and specific peptide inhibitors, the properties of which were reviewed above (see sect. 3.5).

**Figure 5.**
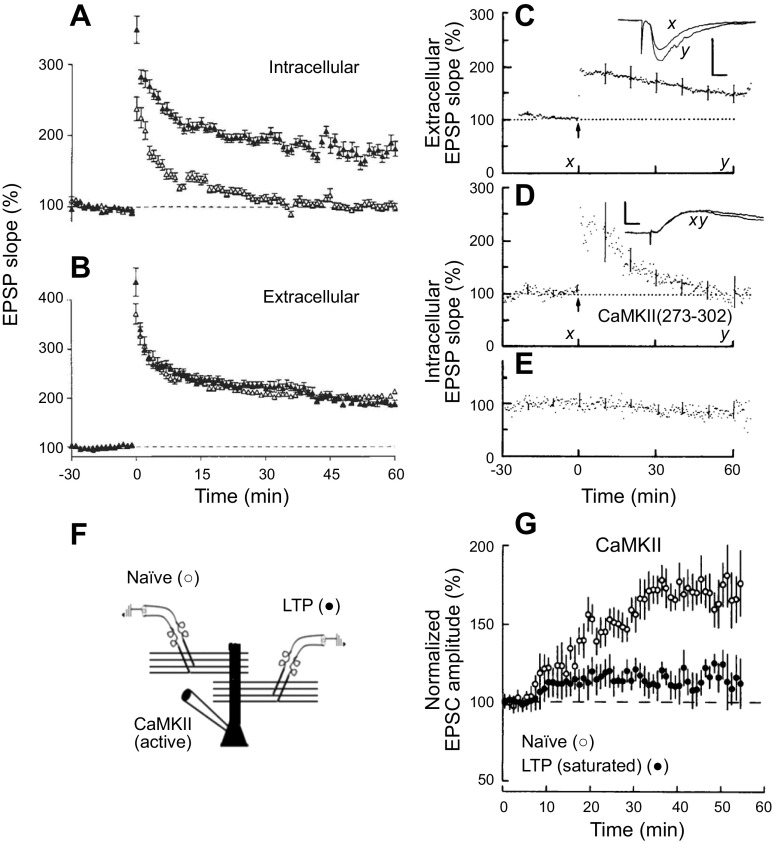
Ca^2+^/calmodulin (CaM)-dependent protein kinase II (CaMKII) is required and sufficient for long-term potentiation (LTP). *A* and *B*: inhibition of CaMKII blocks LTP. Effect of intracellular application of the CaM-binding peptide (CBP) on LTP. *A*: the magnitude of the initial excitatory postsynaptic potential (EPSP) slope in populations of cells recorded with microelectrodes containing 1.1 mM CBP (190 µM: *n* = 11) (open triangles) or the control peptide CTP_2_ (190 µM: *n* = 8) (filled triangles). *B*: the initial slope field EPSP slope recorded in the 2 populations of slices demonstrating that the LTP was essentially identical in the 2 populations (197). *C*: extracellular monitoring shows LTP after tetanic stimulation. *D*: simultaneous monitoring of synaptic potentials with intracellular electrode containing 1.1 mM CaMKII(273–302) shows no persistent potentiation after tetanic conditioning. *E*: transmission in a nontetanized pathway, monitored with the CaMKII(273–302)-containing electrode, is constant throughout the experiment. Error bars indicate SE for representative individual time points. *Insets*: average of 10 consecutive potentials obtained at the times indicated on time axis. Scale bars, 0.33 mV, 12.5 ms (*C*); 5.0 mV, 12.5 ms (*D*) (198). *F* and *G*: constitutively active CaMKII mimics LTP. *F*: diagram showing the recording setup. Two independent pathways are stimulated. In one pathway a saturating level of LTP had been induce. The other pathway serves as a control. *G*: at *time 0* a whole cell recording is made with a patch electrode containing constitutively active CaMKII (truncated). The control pathway shows a robust enhancement, whereas the pathway expressing saturating LTP shows little enhancement (199). Images modified from Refs. 197–199, with permission from *Nature*, *Science*, and *Proceedings of the National Academy of Sciences USA*, respectively.

For physiological studies a variety of approaches has been used to deliver these peptides into cells. These include delivery via the recording pipette, expression of the peptides in cells, or tagging the peptides with membrane-penetrating agents. The authors of this review and their respective collaborators were independently the first to establish in 1989 (published within a week of each other) that infusion of CaMKII peptide inhibitors in the postsynaptic cell blocked LTP (197, 198) (FIGURE 5, *A–E*), providing the first linkage of CaMKII and LTP. In both studies two simultaneous recordings were made: one recorded LTP in a population of neurons with an extracellular field electrode, and the other recorded the response from an individual neuron with an intracellular electrode. When the intracellular electrode contained a CaMKII inhibitory peptide, LTP was blocked in the individual cell but not the population. Numerous studies using peptide inhibitors have confirmed the block of LTP (200–203). Interestingly, the results with the genetic deletion of CaMKIIα are more variable, some showing roughly a 50% block (87, 204–206) but some showing a complete block (121, 207, 208). The reason for the residual CaMKIIα-independent LTP is unclear, although there are multiple CaMKII isoforms and the peptide inhibitors would be expected to block all of them. The residual LTP in the CaMKIIα knockout (KO) may account, at least in part, for the retention of some memory function in this mouse. Perhaps the most compelling results are those in which endogenous CaMKII has been replaced with CaMKII bearing a mutation that prevents autophosphorylation of the autonomy site (T286A, referred to as phosphonull; see sect. 9.2.2). The blockade of LTP in this case is complete. The reason for the variability is not clear. However, when the pharmacological and genetic results are considered together, it seems fair to conclude that NMDAR-dependent LTP is to a very large extent dependent on the activation of CaMKII.

### 5.2. CaMKII and Sufficiency

To test whether CaMKII is sufficient to account for the ability of Ca^2+^ to induce LTP, activated CaMKII was either applied acutely via the recording electrode or expressed in neurons. CaMKII was made constitutively active either by deleting the autoinhibitory segment (truncated CaMKII) (199, 209) or by inserting mutations (e.g., T286D/T305A/T306A) (121, 210). Just using a T286D mutation would be problematic because it generates a constitutively active kinase that then results in the phosphorylation of T305/T306, which can counteract T286D and alter resident time at the synapse (88, 210, 211). Expression of active CaMKII (121, 130–132, 209, 210, 212) or direct delivery into the cell via the recording electrode (199) has repeatedly revealed a roughly two- to threefold enhancement in the AMPAR EPSC but little change in the NMDAR EPSC (130–132). These findings are important for a number of reasons. First, the magnitude of the enhancement is similar to that observed with LTP. Second, the enhancement is selective for the AMPAR EPSC, similar to LTP. Third, these recordings were made 48 h after transfection. This finding indicates that even after tens of hours CaMKII has no effect on presynaptic transmitter release, since there is no change in the NMDAR response. This is seemingly at odds with current models of LTP, e.g., Refs. 19, 213, 214, in which hours after the induction of LTP (late LTP) it is proposed that there is a delayed structural and functional presynaptic modification to match the postsynaptic changes. A photoactivatable form of CaMKII has recently been reported to induce LTP (215). The finding supports the sufficiency of CaMKII in LTP previously demonstrated by introduction of constitutively active CaMKII, although it is possible that the multiple modifications of this CaMKII construct introduce effects unique to the construct.

If the enhancement seen with active CaMKII is related to LTP, CaMKII should occlude LTP, since LTP is saturable. This is indeed the case (199, 209). Furthermore, prior LTP should also occlude the action of CaMKII. Such an experiment is shown in FIGURE 5, *F* AND *G* (199). The experiment involved recording the responses from an individual neuron to two independent pathways (FIGURE 5*F*). A saturating level of LTP was induced in one pathway, while the other pathway served as a control. At this point in the experiment a neuron was recorded at *time 0* with an electrode containing constitutively active (CA) CaMKII (FIGURE 5*G*). The naive control pathway showed a robust enhancement, whereas the response on the pathway expressing LTP was greatly diminished. The above findings indicate that CaMKII both qualitatively and quantitatively precisely mimics LTP and that LTP and CaMKII occlude one another. These findings are important, because they suggest that CaMKII fully accounts for the effects of the rise in Ca^2+^ and that there is no need to postulate a CaMKII-independent parallel Ca^2+^ signaling pathway requirement for LTP. An additional conclusion from these experiments concerns the basis for the saturation of LTP, since overexpression of CA CaMKII causes the same level of potentiation as LTP and LTP occludes the action of CA CaMKII, suggesting that the saturation occurs at a step downstream of CaMKII. It must be stressed that the studies summarized in this section demonstrate the essential role of CaMKII in the induction of LTP. They say nothing about the maintenance of LTP. However, the rest of this review is focused primarily on the role of CaMKII in maintaining LTP.

## 6. CaMKII BINDING TO NMDARs

**Figure 6.**
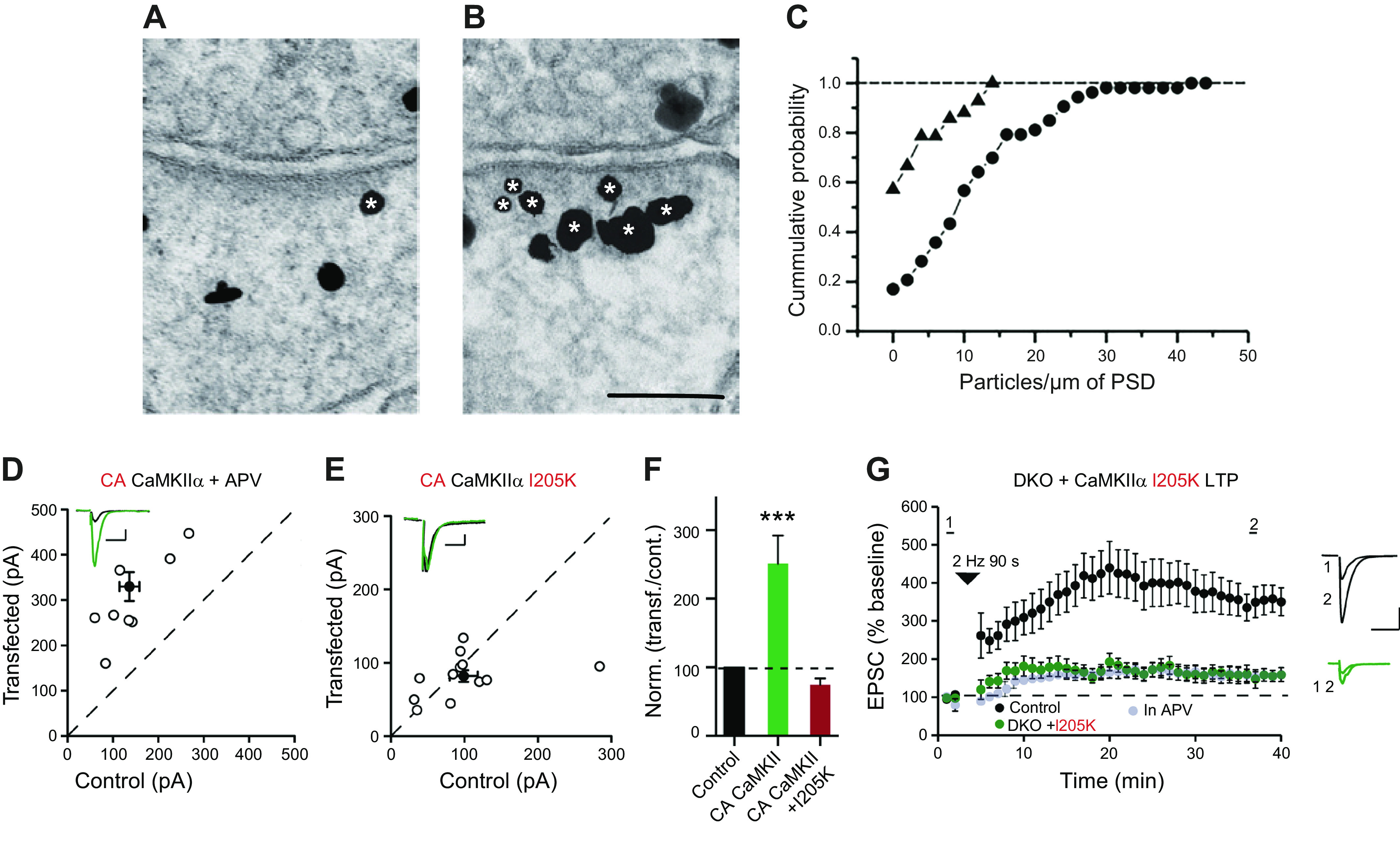
Activity-dependent Ca^2+^/calmodulin (CaM)-dependent protein kinase II (CaMKII) accumulation at the postsynaptic density (PSD) and its actions require binding of CaMKII to *N*-methyl-d-aspartate receptors (NMDARs). *A–C*: chemical long-term potentiation (chemLTP) induction results in persistent accumulation of CaMKIIα at the PSD. *A* and *B*: electron micrographs of hippocampal synapses labeled for CaMKIIα under control conditions (*A*) and 1 h after induction of chemLTP (*B*). Silver-enhanced gold particles appear as irregular black grains. Asterisks indicate grains counted as PSD-associated CaMKII labeling. Scale bar, 100 nm. *C*: cumulative distribution of densities of gold label for CaMKIIα at individual PSDs from slice cultures under control conditions (filled triangles) and after induction of chemical LTP (filled circles) (217). *D*: constitutively active CaMKII (T286D-T305A/T306A) (CA CaMKII) in 2-amino-5-phosphonopentanoic acid (APV) (*n* = 8 pairs) potentiates synapses. *E*: CA CaMKII containing the I205K mutation (*n* = 15 pairs) fails to potentiate synapses. Filled circles indicate mean ± SE. *F*: bar graph of ratios normalized to control (%) summarizing the mean ± SE of α-amino-3-hydroxy-5-methyl-4-isoxazolepropionic acid receptor (AMPAR) excitatory postsynaptic currents (EPSCs) of values represented in *D* and *E* [Mann-Whitney test (***P < 0.0001)]. *G*: cells in which CaMKII has been replaced with CaMKII containing the I205K mutation fail to express LTP. Black filled circles represent control cells, green filled circles represent cells expressing the I205K mutation, and gray filled circles represents experiments carried out in APV (121). *D–F* modified from Ref. 121, with permission from *Nature Communications*.

An interaction between CaMKII and the NMDAR subunit GluN2B was first suggested by the finding that a fragment of GluN2B is a substrate for CaMKII (123). Subsequent studies showed that activation of CaMKII results in the rapid translocation of CaMKII to the PSD (137, 140, 169, 216–220). This translocation involves the binding of CaMKII to the NMDAR (78, 125, 144, 221–224). It should be noted that in one study (225) synapses lacking NMDARs had only a small reduction in the synaptic localization of CaMKII, suggesting contributions of other interacting proteins to its synaptic localization. Of course, not all contributors to CaMKII translocation or synaptic localization need be critical for LTP. The primary interaction in the CaMKII-GluN2B complex is between the CTD, also referred to as C-tail, of the GluN2B subunit and the surface groove in the kinase domain of CaMKII exposed by its activation (61). The persistent accumulation of CaMKII at the PSD following an LTP-inducing stimulus is shown in FIGURE 6, *A–C* (217). The NMDA-induced translocation of CaMKII to the spine/PSD is dramatic, in large excess to the number of NMDARs in the PSD (∼20–30 NMDARs) (see sects. 3.3 and 13.2); thus only a small fraction of the CaMKII holoenzymes recruited to the PSD can be directly bound to GluN2B. We suggest that most of the activated CaMKII may form clusters (see sect. 3.4) that are anchored to the PSD via the few subunits that bind directly to GluN2B. The implication of such a macromolecular complex in information storage is discussed in sect. 13.2.

What consequence does this CaMKII-GluN2B binding have for synaptic transmission? Several approaches have been used to address this question. The first approach removed the GluN2B subunit, either by knockout or knockdown. The results vary from a complete block of LTP (226, 227) to a partial impairment (228, 229). The second approach utilizes mutations in the CTD of GluN2B that cannot bind CaMKII as replacement for the wt (CaMKII L1298A/R1300Q) (120, 121, 230) or either competing with overexpressed full-length constructs (GluN2B R1300S/Q1303D) (231) or competing by expressing just the wt GluN2B CTD (839–1482) (232). The results are highly variable as reviewed in Ref. 233, ranging from complete block of LTP (121, 231) to no block (230). The reason for this variability is unclear.

A third approach takes advantage of a point mutation of CaMKII (I205K), which prevents binding to GluN2B but otherwise reported to function normally (78), keeping in mind that this mutation is expected to also disrupt the binding to densin-180 and α-actinin-2 (234–236) and other PSD proteins. To examine the effects of I205K more directly a comparison was made between the enhancing effect of constitutively active CaMKII (T286D-T305A/T306A) (CA CaMKII) and that with the additional I205K mutation (121). Whereas CA CaMKII had its typical ∼2.5-fold enhancement (FIGURE 6, *D* AND *F*), the CA CaMKII containing the I205K mutation failed to enhance responses (FIGURE 6, *E* AND *F*). Not unexpectedly, LTP was entirely blocked, suggesting that the CaMKII-GluN2B complex is necessary for LTP (FIGURE 6*G*). In this same study (121), in utero electroporation was used to disrupt the CaMKII-GluN2B binding by replacing wt GluN2B with GluN2B(L1298A-R1300Q), which fails to bind CaMKII (120). Replacement with the mutant GluN2B had no effect on NMDAR synaptic currents, indicating that it is functional. Intriguingly, AMPAR responses were reduced ∼50%, similar to the reduction seen by deleting CaMKII, and expressing CA CaMKII failed to enhance AMPAR responses. Furthermore, expressing a constitutively active CaMKII on the background of GluN2B(L1298A-R1300Q) failed to enhance synaptic transmission (121). These results complement previous findings (120) and the results with CaMKII(I205K) and are provocative because, despite being overexpressed at high levels and presumably overcoming any local compartmentalization, this active construct fails to enhance synaptic transmission if just one of its binding targets, GluN2B, is mutated to disable binding. These findings indicate the critical role that binding to the GluN2B CTD has on the synaptic function of CaMKII.

## 7. CaMKII AND THE EXPRESSION OF LTP (CaMKII TARGETS)

### 7.1. Dynamic Properties of the PSD

Our classical view of the synapse is based largely on studies of the neuromuscular junction (NMJ). However, studies of excitatory synapses in the brain, particularly as they relate to LTP, have radically changed our understanding in two fundamental ways. The first is physiological. Based on the NMJ, the postsynaptic element was viewed as a rigid structure. Variations in synaptic strength occurred solely by changes in the probability of transmitter release. As discussed elsewhere (17–22, 237), revelations obtained from studies on LTP indicate that the postsynaptic specialization of central synapses is remarkably dynamic, with a flexibility rivaling that of the presynaptic terminal. Thus, it is now generally accepted that LTP expression is due to the rapid postsynaptic accumulation of AMPARs. This accumulation can occur by two distinct mechanisms. First, there is a population of synapses that lack AMPARs but have a normal complement of NMDARs and are referred to as “silent synapses.” LTP-producing stimuli cause a rapid accumulation of AMPARs referred to as “unsilencing” (238–241). Silent synapses were thought to be limited to young animals (241–244), but a recent elegant study has shown an abundance of silent synapses in mature brain (240). The anatomical substrate for silent synapses is fingerlike dendritic protrusions referred to as filipodia (245), which account for roughly 30% of synapses in the mature brain (240). The second mechanism for the synaptic accumulation of AMPARs involves classical dendritic spines. These synapses contain AMPARs, but LTP-inducing stimuli cause a rapid addition of AMPARs to the synapse (246–250).

The second change in our understanding of synaptic transmission is anatomical. As the dynamic behavior of AMPARs was revealed, new superresolution imaging techniques demonstrated a hitherto unappreciated substructure to the PSD of spine synapses (20, 251–253). NMDARs tend to be concentrated in the middle of the PSD with little overlap with AMPARs (253–255). AMPARs form discrete clusters scattered around the PSD (256–259). Of particular interest is the finding that these clusters are juxtaposed across from presynaptic release site (251, 252, 260). Given the low (mM) affinity of AMPARs for glutamate, this nanocolumn alignment is thought to be necessary for AMPAR activation. It is suggested that activity-dependent synaptic recruitment of AMPARs may involve two steps (259, 261). First, AMPARs are delivered to the PSD, but this is not enough to increase synaptic strength. Second, the newly recruited AMPARs are added to AMPAR clusters in the nanocolumn.

### 7.2. CaMKII Targets

**Figure 7.**
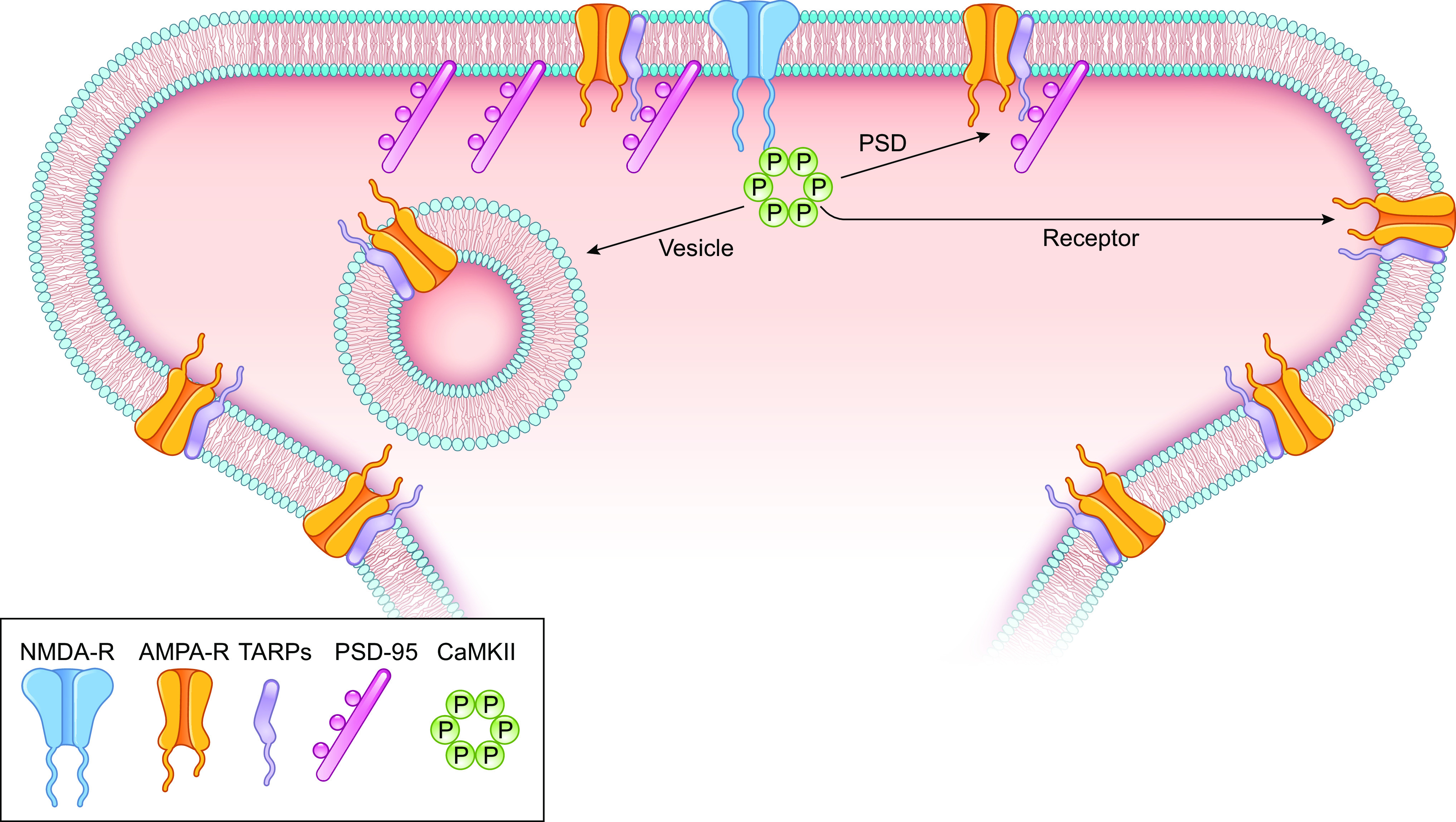
Potential downstream targets of Ca^2+^/calmodulin-dependent protein kinase II (CaMKII) underlying long-term potentiation (LTP). *1*) CaMKII modifies α-amino-3-hydroxy-5-methyl-4-isoxazolepropionic acid receptor (AMPAR)-transmembrane AMPAR-regulatory protein (TARP) complex (Receptor). *2*) CaMKII modifies the postsynaptic density (PSD). *3*) CaMKII modifies the exocytosis of AMPAR/TARP (Vesicle). Only 6 of the 12 subunits are shown for CaMKII. This diagram is not meant to reflect the nanodomain distribution of receptors within the PSD.

As discussed in sect. 6, the formation of a CaMKII-GluN2B complex is essential for LTP, and thus the question arises as to how the increased number of AMPARs and the action of CaMKII are linked. Sects. 7.3–7.5 review and evaluate three distinct models that are proposed to account for the accumulation of AMPARs in LTP. The discussion is divided into three models (FIGURE 7). The first is the receptor-centric model (see “Receptor” arrow in FIGURE 7). In this model CaMKII modifies either the AMPAR and/or the transmembrane AMPAR-regulatory proteins (TARPs). The second model is the PSD-centric model (see “PSD” arrow in FIGURE 7). In this model CaMKII opens or creates slots in the PSD. The third model is the vesicle-centric model (see “Vesicle” arrow in FIGURE 7). In this model CaMKII initiates exocytosis of AMPAR-containing vesicles. It is important to note that these models are not mutually exclusive.

### 7.3. Receptor-centric Model

In this model, the AMPARs and/or auxiliary subunits are directly modified by CaMKII.

#### 7.3.1. AMPARs.

What are the downstream targets of CaMKII that could account for the increase in synaptic strength? With the realization that the increased synaptic transmission involves the recruitment of AMPARs, it was logical to hypothesize that AMPARs are a target of CaMKII (212, 262, 263). Most AMPARs in CA1 hippocampal pyramidal cells are tetraheteromeric assemblies of either GluA1/GluA2 subunits or GluA2/GluA3 subunits with a preponderance of GluA1/GluA2 subunits (264–266). The prevailing model posits that LTP-mediated covalent modification of the intracellular CTD of GluA1 results in the capture of these modified GluA1-containing receptors by preexisting “slots” in the postsynaptic density (PSD) (17, 18, 212). The nature of these slots is unclear, but slots may represent binding sites on postsynaptic scaffolding proteins, such as PSD-95.

Two phosphorylation sites in the GluA1 CTD, S831 and S845, have received most of the attention. Phosphorylation of S831 of heterologously expressed GluA1 by CaMKII increases single-channel conductance (267, 268). However, the degree of phosphorylation of S831 and S845 in vivo is uncertain. It has been reported that the relative abundance of phosphorylated GluA1 is “almost negligible” (Ref. 269, but see Ref. 270). A further issue is that replacement by alanine of either one of these residues does not affect LTP (271); only the double phosphonull mutant partially impaired LTP in adult, but not young, mice (272). These results suggest that, although the direct phosphorylation of GluA1 by CaMKII may play a modulatory role in LTP, it is not essential. Finally, evidence questioning the role of the CTD of GluA1 or GluA2 in LTP comes from molecular replacement studies (273). Experiments in which the CTD of GluA1 is deleted (Refs. 273–275, but see Refs. 276, 277) express LTP that is indistinguishable from that in neighboring control cells. Taken together, these results suggest that the critical target for CaMKII in initiating LTP lies elsewhere.

#### 7.3.2. TARPs.

Evidence has accumulated over the past decade indicating that AMPARs are not stand-alone receptors; rather, they are decorated by numerous types of auxiliary subunits that control the synthesis, trafficking, and biophysical properties of these receptors (278–281). Among these auxiliary subunits, the family of transmembrane AMPAR-regulatory proteins (TARPs) has received the most attention (282). All members of the family contain a cytoplasmic CTD that contains numerous closely spaced serines embedded within an arginine-rich (Arg-rich) region. These serines can be phosphorylated by CaMKII (283). Early studies examining the effects of phosphonull and phosphomimic mutations of the serines suggested a role of these serines in AMPAR trafficking and LTP (283–287). The model based on these studies relies on the interplay between the serines and the Arg-rich motif. Under basal conditions the Arg-rich motif binds to the negatively charged membrane. CaMKII phosphorylation of the serines neutralizes the charge, releasing the CTD and providing access to the PDZ domains of PSD-95 (284, 285, 287).

More recent studies have questioned some of these conclusions. In molecular replacement studies in which GluA1 is tethered to a TARP, the role of serine phosphorylation in AMPAR trafficking and LTP was examined (288, 289). Phosphomimic mutations greatly diminished both the binding of the TARP CTD to PSD-95 and the rescue of AMPAR transmission in AMPAR-deleted cells. These findings suggest that, although phosphorylation of the TARP CTD may release it from the membrane, the diminished binding to PSD-95 dominates. Furthermore, alanine substitutions of the critical serines in the TARP CTD, although reducing AMPAR EPSCs, do not affect the magnitude of LTP (288). Perhaps the differences in results stem from different experimental strategies. Another possibility is that, although the phosphorylation of the TARP CTD might contribute to LTP of heteromeric AMPARs (284, 285), it may play a minimal role during LTP of GluA1 homomeric receptors (289). In summary, although still a matter of debate, the evidence suggests that neither the CTD of AMPARs nor TARPs are likely to be the essential direct downstream target of CaMKII in LTP.

### 7.4. PSD-centric Model

In this model, CaMKII creates slots in the PSD that then capture passively diffusing unmodified AMPAR/TARPs. There are numerous possible targets of CaMKII that could end up creating slots. Since the binding of AMPAR/TARPs to the PSD protein PSD-95 is known to be important for synaptic targeting and LTP (278, 279), controlling this binding and/or the availability of PSD-95 is an obvious scenario. On average there are ∼250 PSD-95 molecules in the PSD (65) and, on average, ∼50 AMPARs, although this number is highly variable (143, 290, 291). Furthermore, AMPARs are highly mobile in the extrasynaptic membrane and can diffuse into and out of the synapse (20). This suggests that many of the PSD-95 molecules are masked and cannot be recognized by AMPAR/TARPs. One possibility is that LTP could unmask PSD-95 molecules, creating new slots. Alternatively, some type of physical rearrangement of the PSD may open up slots.

#### 7.4.1. PSD-95.

CaMKII and PSD-95 are among the most abundant proteins in the PSD. PSD-95 is a member of a family of PSD scaffolding proteins referred to as membrane-associated guanylate kinases (MAGUKs) (292, 293). MAGUKs contain PDZ domains that are responsible for protein-protein interactions. Since there is considerable redundancy in the function of MAGUK proteins we focus on PSD-95, but the other MAGUKs could also be important (259). It is now well established that the delivery of AMPARs to synapses requires the binding of TARPs, via their PDZ binding motifs (PBMs), to PSD-95 (278, 279, 286, 289, 294). Although the CaMKII phosphorylation of TARPs does not appear to be essential for LTP (see above), the next logical candidate is PSD-95. Indeed, PSD-95 can be phosphorylated by CaMKII at S73 (295). Surprisingly, expression of a phosphomimic mutation, S73D, prevents LTP expression (296). However, the results are difficult to interpret, since overexpression of PSD-95 on its own potentiates AMPAR EPSCs and occludes LTP (131, 297, 298) and the S73D mutant causes a similar potentiation. Furthermore, knocking out PSD-95 does not prevent LTP (299). These results make it unlikely that covalent modification of PSD-95 creates the essential slots. However, it is important to keep in mind that redundancy exists among MAGUKs in their role in LTP (259).

#### 7.4.2. SynGAP.

SynGAP is a Ras/Rap GTPase-activating protein that is a major component of the PSD. It has been implicated in synaptic plasticity, and mutations in SynGAP result in intellectual disability in humans (300). It forms a homotrimer, each with a PBM that binds to the PDZ-SH3-GK tandems of PSD-95 with high avidity (301, 302). The multivalent interactions between SynGAP and PSD-95 lead to LLPS (303). SynGAP has a number of intriguing properties suggesting an activity-dependent role in AMPAR synaptic capture. CaMKII phosphorylates SynGAP and decreases its binding to PSD-95 (302, 304). Imaging studies have reported that this phosphorylation triggers movement of SynGAP out of the PSD (304, 305), and this dispersal is required for chemLTP (304). ChemLTP is a procedure in which the slice is bathed with a solution that mimics LTP. Typically this involves removing Mg^2+^ and adding glycine to the perfusate. Furthermore, knockdown of SynGAP results in the accumulation of synaptic AMPARs, which occludes chemLTP (304). A more recent study expanded on these results, proposing that there is a competition between SynGAP and TARPs for binding to PSD-95 (302). The phosphorylation-induced dissociation of SynGAP from the PSD-95 is proposed to free up PDZ domains, creating slots for AMPARs/TARPs (302). The simplicity of the model is seductive. It suggests a link between CaMKII activity and the recruitment of AMPAR to the synapse. However, a critical component of this model is the proposed competition between SynGAP and TARP binding to PDZ1/PDZ2 that is unresolved. Several laboratories have examined SynGAP interactions with PSD-95 using a variety of constructs or forms of SynGAP and PDZ, as well as disparate methods to measure affinities or avidities, thus complicating consensus (301–303). This important hypothesis also needs to be tested in a cellular context.

#### 7.4.3. Rho GTPases/cytoskeleton.

An early enthusiast of spine dynamics was Francis Crick, who posed the question “Do spines twitch?” (306) and predicted the presence of actin in spines. Indeed, spines contain unusually high levels of actin (307–309), and this, coupled with the discovery of structural LTP (sLTP) (247), has focused attention on the possible role of actin in LTP. sLTP is induced by a two-photon glutamate uncaging LTP protocol onto single spines that leads to a rapid and long-lasting enlargement of spines that parallels the enhanced AMPAR responses. sLTP has most, if not all, of the properties of functional LTP, such as synapse specificity and a dependence on both NMDAR and CaMKII. Thus, it has served as a useful proxy for functional LTP. How might the activity-dependent actin remodeling fit into the PSD-centric model of LTP? Actin is known to bind to many PSD proteins (66) including CaMKIIβ (174). Interfering with actin polymerization blocks both sLTP (247) as well as LTP (310). One of the major regulators of actin polymerization is the Rho family of guanine nucleotide exchange factors (RhoGEFs). RhoGEFs catalyze GDP/GTP exchange on small Rho guanine nucleotide-binding proteins (Rho GTPases), which in turn regulate the actin cytoskeleton. Previous studies have shown that the Rho GTPase Rac1 regulates synaptic AMPAR expression (311) and that the Rho GTPases Cdc42 and RhoA are required for LTP and sLTP (312, 313). How might CaMKII engage this pathway? Might RhoGEFs, which are responsible for synaptic Rho GTPase activation, relay the CaMKII signaling, via Rho GTPases, to the changes in the actin cytoskeleton?

The RhoGEFs that have received the most attention are kalirin-7, Trio, and Tiam, which are all expressed in spines and phosphorylated by CaMKII (314–317). Kalirin and Trio serve critical and functionally redundant roles in supporting excitatory synapse structure and function (32, 318). CaMKII phosphorylates Kalirin at T75 (Ref. 315, but see Ref. 317), and this phosphorylation is sufficient to enhance synaptic transmission. Although inhibiting Kalirin function alone has little effect on LTP (318, 319), simultaneously inhibiting CaMKII signaling through Kalirin and Trio eliminates LTP (318). These results suggest that NMDAR-mediated activation of CaMKII induces functional LTP through phosphorylation of Kalirin and Trio.

A recent study has proposed an intriguing role for Tiam1 in sLTP (314). The authors find that Tiam1 has a CaMKII binding domain. This domain is homologous to the GluN2B CTD, and thus the Tiam1-CaMKII complex formation results in constitutive CaMKII activity. CaMKII, in turn, phosphorylates Tiam1. Such a positive feedback loop formed by a reciprocally activating kinase-effector complex enables persistent CaMKII activity for maintaining sLTP. A caveat to this proposal is that the expression levels of Tiam1 in the CA1 region of the hippocampus are very low and deleting Tiam1 has no effect on synaptic transmission (320). However, such a mechanism may well be involved in the dentate gyrus, where Tiam1 expression is high and does contribute to synaptic transmission (320).

In summary, perhaps the strongest case for the requirement of downstream CaMKII targets in sLTP, and likely LTP, is the actin cytoskeleton (30, 313, 321), although the need for phosphorylation of downstream targets is debated (see sect. 9.2). How activity-dependent actin rearrangement fits into the PSD-centric model of LTP is still uncertain. Perhaps actin polymerization during LTP induction causes a structural rearrangement of the existing PSD scaffolding molecules such that slots that were previously inaccessible are now available for AMPAR/TARP interaction. However, actin is largely excluded from the core of the PSD, interacting with such proteins as Shank and SAPAP located in the deeper, cytoplasmic-facing layer of the PSD referred to as the pallium (66, 162, 322). We return to the role of actin in sect. 13.2.

#### 7.4.4. Liquid-liquid phase separation.

As is evident from this PSD-focused section, although CaMKII phosphorylates many dozens of synaptic proteins we still have not definitively established its critical downstream targets. A recent provocative study raises the possibility that CaMKII can initiate a structural rearrangement that assembles critical PSD proteins in vitro without phosphorylating exogenous proteins (323). The authors show that a mixture of the GluN2B CTD and CaMKII undergoes liquid-liquid phase separation (LLPS) when Ca^2+^/CaM binds to CaMKII in either the absence or presence of ATP. The understanding of cellular processes has advanced from binary interactions of enzyme and substrate to amplification of signaling by protein kinase cascades and improved signal specificity and efficiency by protein complexes such as signalsomes and synaptic PSDs. But the notion of protein condensates forming LLPS in regulation of diverse function, including synaptic plasticity, is quite new and not yet fully appreciated by many neuroscientists. Studies in a variety of fields have advanced an understanding of compartments, such as the centrosome and cellular bodies, which can form in the absence of a membrane yet separate themselves from the general pool of soluble proteins (324, 325). These studies can inform our investigation of possible LLPS involvement in signaling at the PSD. Condensates require proteins capable of multivalent interactions to produce a mesh or network that concentrate proteins and drive phase separation. Interestingly, such condensates can persist even with rapid turnover of their proteins. Low-valency proteins such as those interacting with multivalent proteins can be viewed as cargo that moves dynamically in and out of the condensate. Importantly, such cargo can exhibit switchlike partitioning based on availability of scaffold binding sites (325). Such partitioning is also seen in PSD condensates, as exemplified by Arc, which disperses AMPAR/TARPs from the PSD condensate by competing with PSD-95 for interaction with unphosphorylated TARP (326).

**Figure 8.**
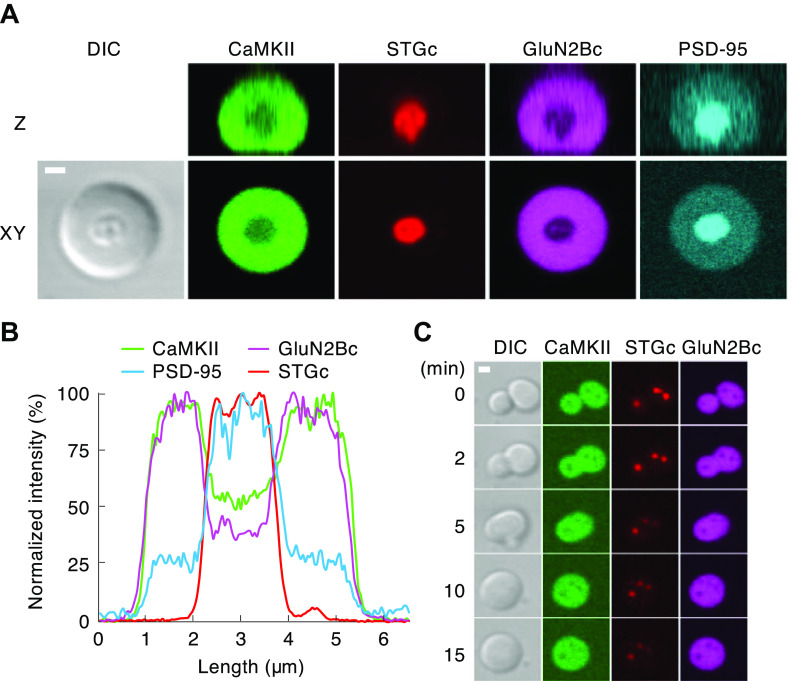
Segregation of α-amino-3-hydroxy-5-methyl-4-isoxazolepropionic acid receptor (AMPAR) and *N*-methyl-d-aspartate receptor (NMDAR) in protein condensate by active Ca^2+^/calmodulin (CaM)-dependent protein kinase II (CaMKII). The assay includes 10 μM PSD-95, 2.5 μM GluN2Bc, 7.5 μM STGc, 10 μM CaMKII, and 10 μM CaM in the presence of Mg^2+-^ATP. *A*: magnification and *Z* projection of single condensates by differential interference contrast microscopy (DIC). Scale bar, 1 μm. *B*: line scanning of *A* in each color channel. *C*: observation of a condensate fusion event. Scale bar, 1 μm. When stimulated with Ca^2+^, PSD-95/STGc formed phase-in-phase while GluN2Bc/CaMKII formed a surrounding phase. This persisted even after addition of EGTA. Modified from Ref. 323, with permission from *Nature Neuroscience*.

The phase separation resulting from GluN2B CTD and CaMKII mixed in the presence of Ca^2+^/CaM remains intact after the removal of Ca^2+^, but only if T286 is phosphorylated. Furthermore, when AMPAR/TARPs (represented by the CTD of the auxiliary TARP subunit Stargazin) and PSD-95 are added to the system, activation of CaMKII in the presence of ATP partitions AMPAR/TARPs and NMDARs into two different phases (FIGURE 8). Remarkably, when the CTD of neuroligin 1 is included, it partitions with the AMPAR/TARPs but not with the NMDARs, reminiscent of nanocolumns in the PSD (251–253) (see sect. 7.1). CaMKII coordinates a variety of cellular functions via phosphorylation of key substrates, but it seems that for regulating the PSD protein assembly in a persistent manner the only necessary phosphorylation is of itself, on T286. As we explore mechanisms for recruiting AMPARs to the synapse it will be important to consider that LLPS may be a general mechanism for assembly of PSDs and that changes in protein expression or phosphorylation can effect a desired change in synaptic strength via modulation of phase separation in the PSD (301).

The condensation of CaMKII and GluN2B CTD has some similarities and differences with the previously described complex that generated a constitutively active CaMKII (78). In both cases addition of Ca^2+^/CaM in the absence of ATP or with the T286A mutant was sufficient for the LLPS or complex formation. However, in the LLPS study, even after long incubation the CaMKII-GluN2B CTD dissipated after removal of Ca^2+^ unless the kinase was allowed to autophosphorylate, albeit slowly. In contrast, the CaMKII-GluN2B CTD interaction and constitutive activity appeared to transition to a stable interaction that was almost as persistent with T286A as with phosphorylated T286. Importantly, although the maintenance of this segregation in the LLPS study required T286 autophosphorylation, it did not require the phosphorylation of any other component of the reconstituted system. In this scenario the autophosphorylation functions to make the docking site for GluN2B persistently available and the CaMKII-GluN2B complex would serve as a hub for further protein complex assembly.

The requirements for T286 phosphorylation in the LLPS but not the biochemical study present two different requirements for maintenance of memory. In the LLPS study, autophosphorylation is necessary for persistence of the condensate. A mechanism for propagation of the autophosphorylated state would then be a requirement for sustained synaptic potentiation. In the binding study, no autophosphorylation is necessary; the transient CaMKII-GluN2B complex transitions into a persistent form that remains constitutively active. In this case there would be no role for T286 phosphorylation to sustain synaptic potentiation. Although there is no mechanistic requirement for T286P to enable a CaMKII-GluN2B interaction in either model, the autophosphorylation probably occurs when ATP is present. Thus, CaMKII is likely to be autophosphorylated in the initial complex. It is essential to learn how closely these in vitro systems model the intact PSD. In contrast to the freely diffusing components in solution, the PSD in intact cells is a highly organized dense thicket of proteins. It is important for the success of this model for the components in the PSD to be mobile. The mobility of these components has been assessed by recovery after photobleaching and single-molecule tracking. These studies show that many synaptic proteins are remarkably dynamic, including AMPARs (237, 249, 327, 328), NMDARs (329), CaMKII (330), and scaffolding proteins, such as PSD-95 (331). Thus, the PSD may not provide a hindrance to LLPS.

### 7.5. Vesicle-centric Model

The premise behind this model is that the number of surface AMPARs for LTP is limiting and that exocytotic membrane fusion delivers the needed receptors to the surface. Numerous studies have shown that blocking postsynaptic exocytosis by a variety of means blocks LTP/sLTP (195, 249, 250, 332–338). Syntaxin (Stx) is a critical component to the SNARE complex, but its role in LTP is controversial. On the one hand Stx-3 is reported to be required for LTP (Ref. 334, but see Ref. 339), while on the other hand Stx-4 is reported to be required for LTP (336, 338). It is unclear what the basis for this disagreement is. Postsynaptic synaptotagin1 and 7 (Syt1/Syt7) have also been reported to be required for LTP (195). Surprisingly, in most of the studies in which blocking exocytosis blocks LTP there is no decrease in basal synaptic transmission, suggesting that there are at least two distinct exocytotic pathways, one for maintaining basal transmission and one dedicated to LTP.

#### 7.5.1. Exocytosis of AMPAR.

By tagging AMPARs with a pH-sensitive tag (SEP-GluA1), the insertion of AMPARs into the surface membrane can be directly visualized during LTP-inducing protocols (249, 250, 335, 337, 338, 340). Additional support for the vesicle-centric model is the finding that AMPAR-containing vesicles (obtained by immunoisolation) are associated with such proteins as synaptotagmin-1 (341), which is involved in LTP (195).

However, several issues must be kept in mind when interpreting these studies. First, it is assumed that surface AMPARs are limiting, and yet it is well established that there is an abundance of extrasynaptic AMPARs. For instance, application of glutamate to somatic nucleated patches, which are devoid of excitatory synapses, generates nanoamps of AMPAR-mediated current (342), consistent with a high density of surface AMPARs. Furthermore, surface AMPARs are highly mobile and proposed to move freely into and out of the synapse (20). Second, immunogold labeling studies find that AMPARs within spines, e.g., in spine vesicles, are scarce, except for large spines containing a spine apparatus (290). Third, most of these studies have tagged GluA1 on the NH_2_ terminus. This tagging severely impacts the trafficking of the GluA1-containing receptor (343, 344). In addition, overexpressing the receptors might cause expression in compartments that do not normally express the receptors. Fourth, in most cases the AMPARs are inserted via exocytosis into the dendritic shaft, some distance from the synapse (220, 249, 250, 337). Fifth, a comparison of the synaptic accumulation of SEP-GluA1 by glutamate uncaging, with and without prior bleaching of surface receptors, indicates that much of the accumulation is due to lateral diffusion and not exocytotic insertion of receptors (250). Consistent with this result is the finding that interfering with the surface diffusion of AMPARs by receptor cross-linking markedly impairs LTP (345–347). Sixth, whereas the recovery from bleaching spine SEP-GluA1 following glutamate uncaging shows a modest dependence on CaMKII, single SEP-GluA1 exocytotic events during glutamate uncaging appear to be independent of CaMKII (250).

Thus, the role of AMPAR exocytosis in LTP remains confusing, especially as it relates to CaMKII. If one accepts that CaMKII is both necessary (43, 121, 207) and sufficient (199, 209, 210) for inducing LTP, then the exocytosis of AMPARs resulting from CaMKII action should be blocked by CaMKII inhibitors. Moreover, the enhancement of transmission by activated CaMKII should be blocked when exocytosis has been blocked. The latter experiment has not been done, but, as discussed, CaMKII appears not to be required for AMPAR exocytotic events (250). There are rather few examples of CaMKII directly initiating exocytosis (348, 349). The above findings are difficult to incorporate into NMDA-dependent LTP.

#### 7.5.2. Exocytosis of other factors.

It is important to note that, although it is assumed that the role of exocytosis is to deliver AMPARs, it is quite possible that exocytosis, perhaps via VAMP2 (350), could be required to deliver some unidentified factor that is necessary for LTP rather than AMPARs. This delivery would have to be dependent on CaMKII. For instance, this factor might provide bridging of the amino-terminal domain of AMPARs to transsynaptic adhesion proteins (343, 344, 351). Another factor that could be released by exocytosis is brain-derived neurotrophic factor (BDNF) (352, 353). It has been proposed that CaMKII-dependent release of BDNF-containing vesicles initiates an autocrine feedback, in which BDNF activates TrkB receptors that in turn trigger actin cytoskeleton remodeling. There is support that CaMKII can mediate the activity-dependent exocytosis of BDNF (349), but the linkage between TrkB activation and the synaptic accumulation of AMPARs remains unclear. Moreover, although it is claimed that BDNF enhances synaptic transmission (354), this finding has been challenged (355). Furthermore, it has been reported that, although BDNF is required for an NMDAR-independent presynaptic form of LTP, it is not required for NMDAR-dependent LTP (196). To summarize, there is general agreement that exocytosis is required for LTP, consistent with the vesicle-centric model. However, further work is required to determine whether the vesicular cargo is indeed AMPARs or some other factor.

### 7.6. Summary

A great many downstream targets have been proposed for the action of CaMKII. Except for the role of Rho GTPases, which appear to be essential for LTP, many of the proposed targets may play a modulatory role but are not essential for LTP. In the case of the vesicle-centric model of LTP, it seems clear the LTP requires an exocytotic event. Since CaMKII is sufficient for LTP, it predicts that CaMKII activation initiates the exocytosis, but this has yet to be demonstrated. To date, the strongest case can be made for the PSD-centric model. The evidence is compelling that CaMKII initiates a dramatic rearrangement of the cytoskeleton that is required for sLTP and most likely for LTP. Finally, accumulating evidence supports the possibility that the CaMKII-GluN2B complex serves as a central organizing hub. The parallels between the postulated reorganization of the PSD following LTP and the behavior of the critical components in solution as assayed by LLPS are most appealing for the PSD-centric model.

## 8. SYNAPTIC TRANSMISSION IS MAINTAINED BY THE CONSTITUTIVE ACTION OF CaMKII

As discussed above, early on it was shown that CaMKII is both necessary and sufficient to initiate LTP, thus solidifying their association. However, if CaMKII is to underlie the maintenance of LTP and serve as a memory trace, there are two essential predictions. First, LTP acquired when the animal was alive should leave a lasting trace, i.e., the ensemble of potentiated synapses would contribute to baseline synaptic transmission (44, 356). Second, if the maintenance of LTP requires CaMKII, then blocking CaMKII after the induction of LTP must reverse the potentiation. The failure to satisfy either of these predictions during the past two decades caused extreme strain on the hypothesis that CaMKII plays a role in the maintenance of LTP and synaptic memory. In this section we address the issue of CaMKII and baseline synaptic transmission, and in sect. 9 we address the role of CaMKII in the maintenance of LTP. Baseline synaptic transmission must reflect the composite response of synapses that have been potentiated, depotentiated, or not yet modified. One test of the prediction that CaMKII has a role in creating a lasting memory trace is to determine whether CaMKII inhibition or genetic deletion reduces baseline transmission. However, as mentioned above, most genetic deletion studies have failed to observe effects on baseline synaptic transmission (Refs. 43, 204, 205, but see Ref. 206). In addition, pharmacological inhibition of CaMKII activity has generally been reported not to affect synaptic responses (198, 200–203, 357, 358).

Recent findings have prompted a reevaluation of CaMKII’s role in baseline synaptic transmission. The first evidence that CaMKII contributes to synaptic transmission came from expression of peptide inhibitors (150, 154). By comparing synaptic responses between a control cell and one expressing the inhibitory peptide, it was found that the AMPAR response, but not the NMDAR response, was reduced by 50%. More recent experiments with either RNAi (211) or CRISPR (121) confirmed the role of CaMKII in maintaining synaptic responses. Although most studies showing a decrease in baseline transmission are based on single-cell gene deletion, this cannot account for the difference, because a similar depression is observed with bath application of inhibitors and field potential recording. It should be mentioned that field potential input-output curves used in many of the knockout mouse studies are not as sensitive as paired recordings from control and transfected cells used in the more recent investigations.

### 8.1. Application of CaMKII Inhibitors Reduces Synaptic Transmission

**Figure 9.**
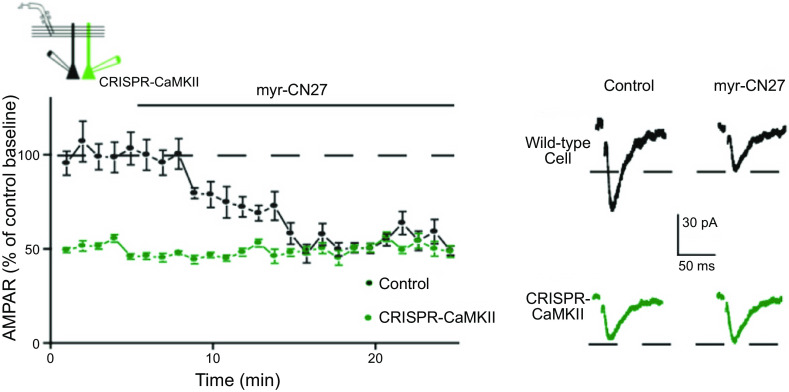
Blocking Ca^2+^/calmodulin-dependent protein kinase II (CaMKII) selectively depresses α-amino-3-hydroxy-5-methyl-4-isoxazolepropionic acid receptor (AMPAR) excitatory postsynaptic currents (EPSCs). *Inset* shows the recording arrangement (black cell is control; the green cell lacks CaMKII). Time course of the effect of myr-CN27 (1 µM) on AMPAR EPSCs in wild-type (wt) cells (black circles) and simultaneously recorded CRISPR-CaMKIIα-transfected cells (green circles), normalized to wt baseline (from culture slices). Although myr-CN27 inhibited AMPAR EPSCs in wt cells, it had no effect on CRISPR-CaMKIIα-transfected cells (*n* = 6, *P* > 0.05, 2-tailed Wilcoxon signed rank test). Image from Ref. 361, with permission from *eLife*.

To test whether the constitutive CaMKII activity reflects LTP acquired before slice preparation, it is necessary to have rapidly acting and reversible inhibitors of CaMKII. As discussed above (see sect. 3.5), two classes of peptides have been developed: those derived from the autoinhibitory domain of CaMKII (e.g., AIP) and those derived from an endogenous CaMKII inhibitory peptide referred to as CaMKIINtide (e.g., CN compounds) (145). Both classes of peptides are thought to bind along the substrate binding pocket (61, 62). However, for these peptides to be useful they need to act rapidly and reversible, and yet peptides do not effectively cross cell membranes. Thus, more recent studies have coupled these peptides to cell-penetrating agents. tatCN21 (154, 202, 359), antCN27 (151, 360), myr-CN27 (150, 361), and myr-AIP (361) all reduced synaptic transmission. It is unclear why tatCN21 and antCN27 cause a substantial reversible nonspecific presynaptic depression (154, 359, 360), which is then followed by a lasting depression of postsynaptic responses. myr-CN27 and myr-AIP lack this nonspecific effect, since the depression of AMPAR EPSCs is not accompanied by any change in the NMDAR EPSC, thus ruling out any nonspecific presynaptic action. Application of myr-CN27 causes a highly reproducible inhibition that takes tens of minutes to stabilize (FIGURE 9) (361). In these experiments simultaneous recordings were made from a cell in which CaMKII had been deleted with CRISPR (see diagram in FIGURE 9). The synaptic transmission in cells lacking CaMKII was ∼50% of that of the control cells, consistent with a maintenance role of CaMKII. Importantly, although myr-CN27 had its usual depressant action on control cells, it had no effect in cells lacking CaMKII, indicating the specificity of the peptide. These results also show that 1 µM myr-CN27 fully inhibits CaMKII. Additional experiments show that there is no recovery after washout of the peptide for at least 1 h. An inhibition of synaptic transmission with identical properties including magnitude, selectivity, and time course was found with myr-AIP (361). The slow time course is not due to slow access of the inhibitors to the site of action, since paAIP2, a photoactivatable peptide inhibitor (203) that acts within seconds of light exposure, has the same slow time course (361). The slow rate therefore reflects the rate at which the synaptic actions of CaMKII are reversed, e.g., displacement of CaMKII-GluN2B binding, phosphatases, etc.

### 8.2. Origin of Constitutive CaMKII Activity

In addressing the origins of the “constitutive” CaMKII activity and whether it represents a synaptic memory trace, there are three sources to consider. First, does isolated CaMKII (unphosphorylated) in the absence of Ca^2+^/CaM have any intrinsic activity? As discussed in sect. 2.1 this is most unlikely. Second, is the observed constitutive activity detected in neurons generated by ongoing stimulation either by spontaneous NMDAR activity or action potentials or by resting levels of Ca^2+^ in the cell? This possibility has been addressed in several experiments. Chelating postsynaptic Ca^2+^ has no effect on this CaMKII constitutive activity (361). If elevated Ca^2+^ is responsible for the CaMKII activity, then there should be a full recovery of synaptic responses after the transient inhibition of CaMKII activity. This is not the case. After the transient inhibition of CaMKII with the photoactivatable inhibitor (paAIP2) (203), there is no recovery (361). Thus, the evidence indicates that baseline Ca^2+^ and spontaneous Ca^2+^ transients do not activate CaMKII. The unusually high Hill coefficient of ∼6 for CaMKII (108) and the high requirement for Ca^2+^ (half maximal of ∼1.6 µM at saturating CaM) compared to the resting level of Ca^2+^ (20–50 nM) (109, 110) ensures maintenance of the synapse specificity and Hebbian nature of LTP (19).

### 8.3. Experimental Manipulations of Constitutive CaMKII and LTP Mirror One Another

We are therefore left with the third possibility, that baseline autonomous activity is acquired during prior LTP. Do experimental manipulations of either LTP or CaMKII show corresponding changes in CaMKII or LTP, respectively, in a manner supporting a central role of constitutively active CaMKII maintenance of LTP? *1*) Peptide inhibition of CaMKII has no effect on the NMDAR EPSC in accord with an LTP mechanism, since LTP is expressed predominantly on AMPAR ESPCs (19). *2*) Deleting NMDARs in individual neurons or replacing GluN1 with a pore-dead mutant in utero, thus preventing NMDAR-dependent LTP, should prevent generation of constitutive CaMKII activity. This indeed is the case (361). *3*) LTP is saturable. Thus, if one were to transiently inhibit CaMKII and reduce baseline transmission, the magnitude of LTP should be larger. Again, experiments confirm this prediction (361).

The notion that the constitutive action of CaMKII contributes to synaptic transmission is provocative. It has generally been assumed that the synapses studied in a hippocampal slice, in which much of the afferent drive from multiple inputs has been removed in the slicing, are at a “ground state” or “basal state.” However, the findings reviewed here indicate that the baseline synaptic currents we measure are actually maintained by a persistent enhancement acquired before slicing. Thus, it seems more appropriate to characterize the responses recorded in slices as being “baseline” rather than being “basal.”

What accounts for the constitutive CaMKII at the level of the synapse? This question in turn raises a fundamental issue in the LTP field: at individual synapses, is LTP all or none or graded (362, 363)? The LTP-induced unsilencing of synapses (238–240) certainly indicates that LTP can be all or none. However, there is evidence that synapses that already contain AMPARs can undergo LTP. For instance, the size of “quantal” miniature EPSCs increases after LTP (364, 365). Furthermore, two-photon glutamate uncaging experiments on single spines indicate that synapses that already express functional AMPARs can be further enhanced by inducing LTP (246–248). Thus, it seems reasonable that based on the history of a synapse, pyramidal cells presumably contain a mixture of synapses that have experience LTP and those that have not. Remarkably, the magnitude of the effect of deleting or pharmacologically blocking CaMKII is highly reproducible when analyzed among a population of synapses. In young slices, adult slices, and slice culture the reduction is ∼50%. This suggests that the overall magnitude of the LTP that a pyramidal cell expresses is tightly regulated.

Is the contribution of CaMKII to synaptic transmission a net gain that simply adds onto the preexisting excitatory drive of the cell? If this were the case, it has been argued that the system would be unstable and quickly saturate (366–371). There are at least three ways that a cell is postulated to guard against such a catastrophic outcome. Two are acute, whereas the third is on a longer timescale. First, it has been reported that LTP is often accompanied by a depression in neighboring nontetanized synapses (372, 373), which would tend to renormalize excitatory drive onto the neuron. Second, NMDAR-dependent long-term depression (LTD) depresses synaptic transmission and can reverse established LTP (374, 375) (see sect. 9.2.3). Such bidirectional control had long been postulated as necessary for a mnemonic device to prevent saturation. The third mechanism, homeostasis, occurs on a slower timescale of many hours or days (368). In this case the cell senses the excess level of activity and through a cellwide mechanism scales down all synapses in a multiplicative manner to restore excitatory drive to a set point. Thus, the CaMKII “memory” is presumably imbedded in an ongoing dynamic regulatory process that maintains overall stability of the neural network. Perhaps this homeostatic mechanism is involved in maintaining the overall level of LTP on a pyramidal cell at roughly 50%.

## 9. CaMKII AND THE MAINTENANCE OF LTP

We imply in sect. 8 that the reduction in synaptic transmission following inhibition of CaMKII reflects a reversal of prior LTP. In addition, LTP-inducing stimuli result in the persistent accumulation of CaMKII in the PSD (FIGURE 6, *A–C*) (217). However, numerous previous studies have failed to reverse established LTP (Refs. 198, 200–203, but see Ref. 357). Thus, the field has essentially remained at an impasse for decades. Recent studies have made inroads on this impasse. As discussed above, if the contribution of constitutive CaMKII to synaptic transmission reflects prior LTP when the animal was alive, then it should be possible to reverse LTP with these same CaMKII inhibitors.

### 9.1. CaMKII Inhibitors Erase LTP

Experiments in which CN21 or CN27 has been made membrane permeable, either with antennapedia (AntCN27) (151) or with tat (tatCN21) (154), provided indirect evidence that CaMKII inhibitors might reverse LTP. After the induction of saturating LTP, CN21/CN27 was transiently applied. An hour after washout of the peptide, LTP was reintroduced. Whereas no LTP could be induced with a scrambled peptide, because LTP remained saturated, some LTP could be induced after CN21/CN27, suggesting that the peptide partially reversed (unsaturated) the LTP maintenance process. Unfortunately, the strong nonspecific effects of AntCN27 and tatCN21 during the application (see above) precluded directly recording the reversal of LTP.

**Figure 10.**
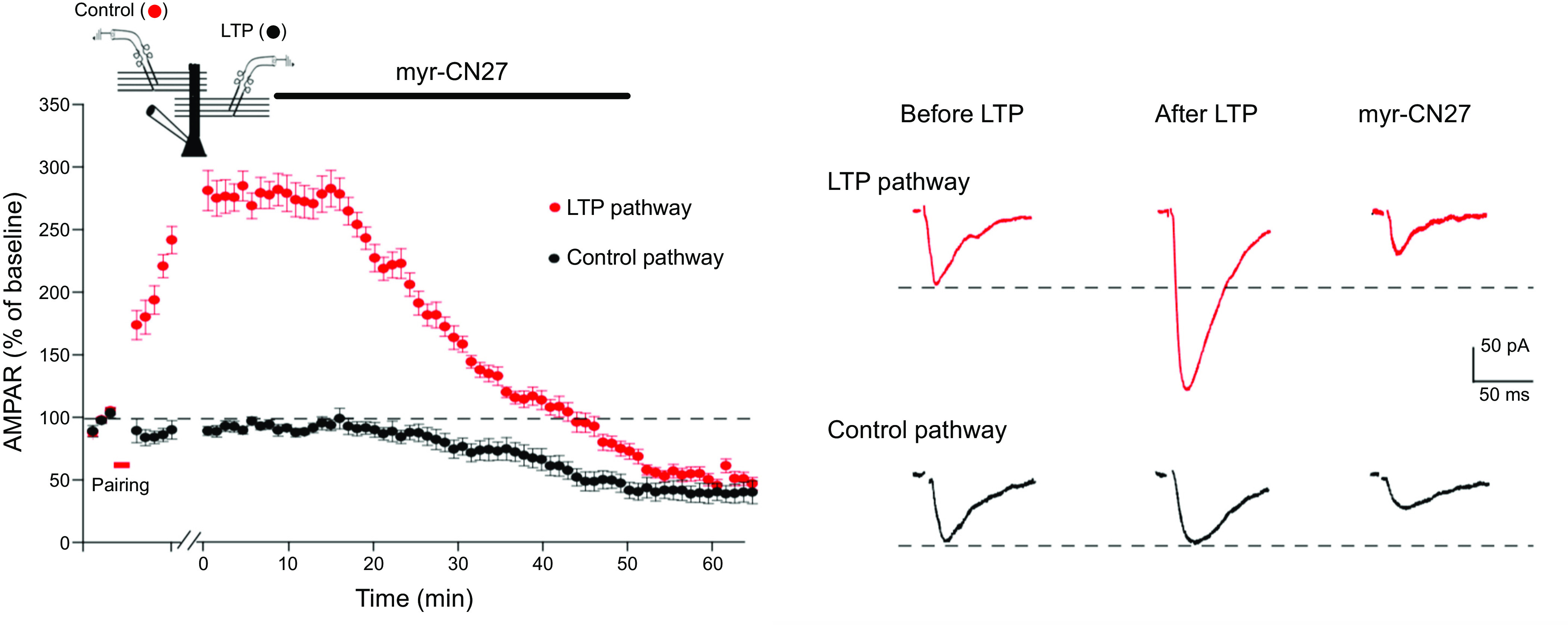
Blocking Ca^2+^/calmodulin-dependent protein kinase II (CaMKII) fully reverses established long-term potentiation (LTP). Diagram shows the two-pathway experimental setup. To record the response from two independent pathways, two bipolar stimulating electrodes were positioned to either side of the recorded cell with a distance of ∼100 μm. Stimuli were applied alternately every 20 s. The summary graph shows that myr-CN27 reduced the control pathway (black circles) 50% while completely reversing LTP (red circles) (difference between control and LTP pathway at 60 min; *n* = 11, *P* > 0.05, 2-tailed Wilcoxon signed rank test). Responses are means ± SE. *Right*: sample traces showing the effect of myr-CN27 on α-amino-3-hydroxy-5-methyl-4-isoxazolepropionic acid receptor (AMPAR) excitatory postsynaptic currents (EPSCs) in control and LTP pathways. LTP is induced by 2-Hz stimulation for 90 s while holding the cell at 0 mV (361).

Because this “erasure experiment” (Lisman’s terminology) is so critically important, it has recently been repeated using myr-CN27, which lacks the nonspecific effects of antCN27 or tatCN21 (361). Thus, it should be possible to directly record the erasure of LTP (FIGURE 10). In these experiments two independent pathways were activated and the responses recorded in a single cell. LTP was established on one of the pathways while the other served as a control. After LTP stabilized, myr-CN27 was applied. LTP was fully reversed and the responses converged with the responses in the control pathway, which, as expected, were reduced by ∼50%. These findings demonstrate that CaMKII is required for maintaining LTP. The failure of previous experiments to reverse LTP may be due to the duration of peptide application, the concentration of the peptide, and the nature of the peptide and penetrating agent.

### 9.2. CaMKII Binding to GluN2B vs. T286 Phosphorylation

The use of currently available CaMKII inhibitory peptides cannot definitively distinguish between blocking kinase activity and the binding of CaMKII to GluN2B, which is required for its synaptic action, since these peptides block both. When discussing the consequences of Ca^2+^/CaM binding to CaMKII, there are two steps that need to be considered. The first step concerns the effect that Ca^2+^/CaM binding has on CaMKII-GluN2B binding, and the second step concerns the properties of the CaMKII-GluN2B complex after the dissociation of Ca^2+^/CaM.

#### 9.2.1. Ca^2+^/CaM binding to CaMKII initiates binding to GluN2B.

The first step involves Ca^2+^/CaM peeling away the autoinhibitory segment from the kinase domain. This results in the phosphorylation of T286 and also the binding of CaMKII to GluN2B even without prior autophosphorylation, both of which maintain CaMKII in an active state (64, 78, 119–121, 221). A critical question is what role, if any, T286 phosphorylation plays in the memory function of CaMKII. It is well established that the Ca^2+^/CaM-dependent binding and phase separation (LLPS) of CaMKII to GluN2B can occur in the absence of enzymatic activity (169, 323, 376). As long as Ca^2+^/CaM is present, binding and phase separation are maintained even with a phosphonull mutant of T286 (T286A) (78, 169, 323, 377) or with a kinase-dead mutant of CaMKII (169, 323, 378, 379), although the binding of CaMKII to GluN2B is modestly enhanced by T286 phosphorylation (378). Taken together, these studies indicate that the Ca^2+^/CaM-dependent binding and phase separation of CaMKII to GluN2B does not require the autophosphorylation that normally accompanies activation.

#### 9.2.2. The nature of the CaMKII-GluN2B complex after the dissociation of Ca^2+^/CaM.

We now turn to the second step. Is autophosphorylation required for stabilizing the CaMKII-GluN2B complex and for LTP? The important question is whether T286 phosphorylation is necessary for the continued binding to GluN2B and for the action of CaMKII in LTP. Informed readers have their choice of data suggesting that the answer is no. First, a biochemical study demonstrated that in the absence of added ATP, and with sufficient time, the binding of CaMKII to GluN2B persists after the removal of Ca^2+^/CaM (78). Second, spine CaMKII activity as monitored with fluorescence resonance energy transfer (FRET) imaging (188) during LTP only remained elevated for ∼1 min after the induction of LTP, suggesting that conformational changes associated with CaMKII activation due to either Ca^2+^/CaM or autophosphorylation are transient. Such a conclusion is tempered, however, if the bulk of the signal is not from CaMKII complexed with GluN2B. Indeed, based on quantitative estimates of CaMKII distribution in spines (380), it was proposed that the active CaMKII bound to the NMDAR was small (∼1%). Another possible concern with these experiments is that the Camuii FRET, which measures the degree of separation of the NH_2_ and COOH terminals of CaMKII, does not directly measure kinase activity. Third, as discussed in sect. 8, concentrations of CaMKII inhibitors that block the induction of LTP failed to affect maintenance (Refs. 198, 200–203, but see Ref. 357). The higher concentrations of inhibitor that do reverse LTP (154, 361) could result from the displacement of GluN2B bound to CaMKII. The extreme view in the no autophosphorylation camp is that the kinase need only complex with GluN2B upon Ca^2+^/CaM binding and be followed by a change in the interaction that makes it persist even when the Ca^2+^ level returns to baseline. In this scenario, neither autophosphorylation nor even CaMKII catalytic activity is proposed.

Countering the evidence arguing against a requirement for T286 phosphorylation in maintaining the CaMKII-GluN2B complex for LTP is a wealth of data for an essential role for T286 phosphorylation. Foremost are data, now long accepted, that NMDAR-dependent LTP is greatly impaired when T286 is replaced by T286A (43, 121, 381–384). Despite these findings suggesting a critical role of T286, it should be noted that Chang et al. (94) reported that LTP can be expressed in the absence of T286 phosphorylation. Furthermore, T286A translocated to the synapse after neuronal stimulation dissociates much more rapidly upon the removal of Ca^2+^ than wt CaMKII. This suggests a need for T286 phosphorylation for persistence of a CaMKII-GluN2B complex (88, 169). In support of these results, both sLTP (379) and LTP (121, 379) are greatly diminished in a CaMKII-dead mutant (K42K or K42M), despite its translocation.

T286 phosphorylation plays a role in the maintenance of liquid-liquid phase separation (LLPS) (323). As discussed above, although the initial formation of CaMKII-GluN2B condensates by Ca^2+^/CaM can occur without phosphorylation, kinase activity, and in particular T286 phosphorylation, is required for the persistent maintenance of the condensates in the absence of Ca^2+^/CaM. Further support for the role of T286 phosphorylation in maintaining the condensates and the binding of CaMKII to GluN2B in the absence of Ca^2+^/CaM is the finding that phosphatase PP2A rapidly dephosphorylates CaMKII, which results in the dissolution of the CaMKII-GluN2B complex (161).

#### 9.2.3. The role of phosphatases in the synaptic action of CaMKII.

A challenge for any model based on autophosphorylated CaMKII is how the phosphorylated state of the kinase and its targets can be maintained long term in the presence of phosphatases and the absence of Ca^2+^ stimuli. How can active CaMKII avoid or overcome the action of phosphatases PP1 and PP2A in the spine (64, 385–387)? Three proposals have been advanced. First. if a substantial number of subunits in a holoenzyme are constitutively active, the rate of autophosphorylation is proposed to exceed the rate of dephosphorylation (64). Second, it is proposed that when Ca^2+^/CaM is bound to CaMKII it competes with and limits the binding of phosphatases to CaMKII (388), although this is less likely to occur at basal Ca^2+^. Third, evidence suggests that the functional pool of CaMKII that is bound to GluN2B is sterically protected from the action of phosphatases (26, 389, 390). It is interesting to speculate and test whether LLPS provides a novel mechanism for creating distinct compartments for CaMKII and phosphatases such as PP1 and PP2A. Phosphatases function in association with numerous synaptic binding partners, often linked to the synaptic cytoskeleton, that can control their localization and therefore access to substrates (391). Thus, in addition to any hypothesized steric factors that shelter T286P from phosphatases, the general scheme of LLPS may provide ways to restrict access of phosphatases to specific substrates such as CaMKII (T286P).

These proposals suggest that under baseline conditions the balance between phosphorylation and dephosphorylation favors phosphorylation. In support of such a balance, it has been reported that PP1 inhibition facilitates the induction of LTP, by promoting activation of CaMKII (392). However, it has long been established that synaptic activity can engage phosphatases. Low-frequency prolonged synaptic stimulation evokes an NMDAR-dependent LTD (374, 393), and blockade of phosphatases by Calyculin A enhances AMPAR responses but not NMDAR responses (Ref. 394, but see Ref. 395). In a series of classic studies it was found that LTD is induced by the activation of a high-affinity Ca^2+^ cascade resulting in the activation of PP1 (395–397). In addition, PP1 has been shown to enhance dissociation of autophosphorylated CaMKII from the PSD (386). Thus, the finding that constitutive CaMKII activity contributes to baseline synaptic transmission provides a substrate for the action of PP1, causing a depotentiation, i.e., LTD.

It is important to mention that additional forms of NMDAR-dependent LTD have been proposed, which differ mechanistically from the simple phosphatase model. One form requires glutamate binding to the NMDAR but is independent of ion flux (398, 399). Another form consists of the competition of DAPK1 (death-associated protein kinase 1) with CaMKII for binding to GluN2B (400). It is proposed that phosphatase activation results in DAPK1-mediated suppression of CaMKII-GluN2B binding. It has also been reported that during LTD-inducing conditions autonomous CaMKII selectively phosphorylates S567 on the GluA1 AMPAR subunit to reduce its synaptic localization (401). Finally, another proposal also focuses on CaMKII and the phosphorylation of T305/T306, which selectively promotes LTD (402). Evidence is presented that T305-P/306-P directly contributes to curbing the level of Ca^2+^-independent autonomous activity. There has been less of a mechanistic focus on LTD compared to LTP, and we are therefore left with a multitude of scenarios leading to LTD, which raises many questions. For instance, few attempts have been made to understand how these different forms can be selectively engaged. This is especially the case in which CaMKII, in addition to LTP, is required for inducing LTD. Do they coexist at the same synapse? If so, are they entirely independent or do they interact?

In summary, although the issue is not entirely settled, the preponderance of evidence favors a role of T286 phosphorylation and CaMKII autonomy in the prolonged physiological action of CaMKII. It is proposed that although autophosphorylation is not required for the formation of the CaMKII-GluN2B complex, it is critical for its stability and maintaining the potentiated state. It should be emphasized that T286 phosphorylation in maintenance allows for bidirectional plasticity in the form of LTD, in which phosphorylated T286 is an obvious target of PP1. The existence of LTD avoids the problem of saturation, which could be essential for a mnemonic device (366–371).

## 10. CaMKII AND PROTEIN TURNOVER

A fundamental question in the field of neuroscience is how memories outlast the lifetime of the molecules that encode them. Although memories can last for the entire span of an animal’s life, most synaptic proteins generally have half-lives of 2–5 days (403, 404). In particular, CaMKII is known to turn over rapidly, with a half-life of 2–4 days (403–405). A possible solution to this problem was proposed at a theoretical level by a two-step model (7, 8). The first requirement is a multimeric protein capable of intermolecular phosphorylation. As reviewed, CaMKII fully satisfies this requirement. The second requirement is that subunits from newly synthesized “naive” holoenzymes can exchange into the active multimeric protein and be phosphorylated by the active subunits in the holoenzyme, thus perpetuating the phosphorylation (memory). An alternative model for propagating CaMKII activity is that an activated holoenzyme might phosphorylate an inactive holoenzyme. However, biochemical experiments have argued against such a mechanism in perpetuating the phosphorylation. Most studies do not find much interholoenzyme autophosphorylation (although see Ref. 81 and discussion below). A further complication for either of these possibilities is that it appears that the subunit being phosphorylated apparently would need to have Ca^2+^/CaM bound, i.e., it does not significantly occur on inactive subunits (13, 15, 82, 83, 406).

**Figure 11.**
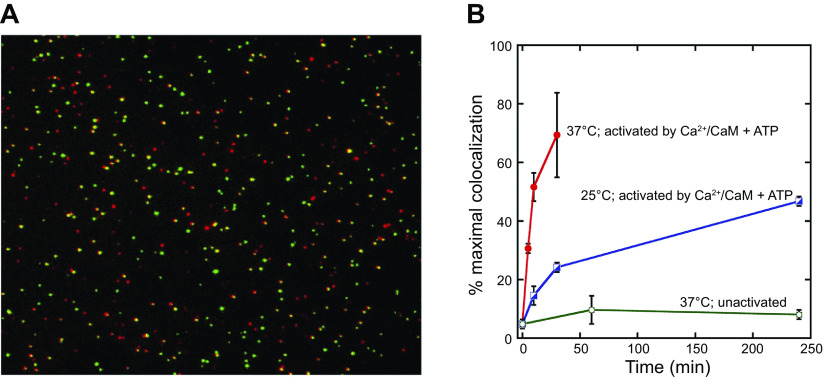
Single-molecule assay for subunit exchange reveals activation-dependent subunit exchange. *A*: a representative single-molecule total internal reflection fluorescence (TIRF) image, with red and green channels overlaid. *B*: the rate of increase in colocalization is significantly faster at 37°C (red) compared to 25°C (blue) when Ca^2+^/calmodulin (CaM) and ATP are added. At 37°C, the unactivated sample (i.e., with no addition of Ca^2+^/CaM and ATP) shows only a low level of exchange even at long time points (green). Image from Ref. 406, with permission from *eLife*.

Recent biochemical evidence suggests that CaMKII may also satisfy this second requirement, i.e., for subunit exchange (28, 406–408). With the use of total internal reflection fluorescence (TIRF) microscopy to track single molecules of CaMKII labeled on their catalytic domains with fluorescent dyes, it was found that activation of two homogeneous populations of CaMKII holoenzymes each with a distinct fluorophore triggers substantial colocalization consistent with the exchange of subunits (FIGURE 11) (406). This subunit exchange process requires CaMKII activation and is initiated after phosphorylation of T305/T306 in the CaM footprint, which blocks rebinding of CaM and frees the CaM-binding element to interact with the hub of the holoenzyme (407). A basic portion of the CaM binding domain exposed after activation and free of CaM may dock at the intersubunit interface of the hub and act as a wedge that breaks integrity of the hub and facilitates release of dimeric units (407, 408). Two requirements must be met for this to help propagate the activity to new naive kinase. The first is for exchange of subunits (perhaps dimers) between holoenzymes. Indeed, it was found that mixing inactive holoenzymes with a holoenzyme made active with the T286D mutation resulted in subunit exchange and colocalization in the same holoenzyme of active and inactive subunits in the absence of Ca^2+^/CaM. But if release of dimers requires CaMKII activation, how are inactive dimers generated? For active holoenzymes, this dimer subunit shedding has been worked out. However, it is less clear how an inactive holoenzyme sheds a dimer. Perhaps the shedding of dimers from inactive holoenzymes does occur to a minor extent. Since the spine contains a great excess of inactive holoenzymes, a low level of shedding would be enough for incorporation into the few active holoenzymes. Alternatively, an inactive dodecamer would allow for insertion of an active dimer to make a transient tetradecamer, which would then phosphorylate inactive subunits. Another possibility, at least in vivo, could rely on the well-established finding that CaMKII is synthesized locally in dendrites (98, 409, 410). Assuming that the assembly of CaMKII holoenzymes involves transient free dimer subunits, perhaps these inactive dimers could be a source for subunit exchange. Finally, the assembly of new holoenzymes could incorporate shed active dimers.

The second requirement for propagation of the autophosphorylated and active kinase is for the naive dimers that achieve exchange into an autonomously active holoenzyme to be autophosphorylated on T286 in the absence of Ca^2+^. How this can be achieved is not apparent from the literature. Although autophosphorylation does occur by transphosphorylation between subunits within a holoenzyme, the substrate subunit needs to be bound to Ca^2+^/CaM because that is what exposes T286 (82, 83), thus challenging the model of perpetuating CaMKII activity. These original assays were carried out at low concentrations over brief time courses that clearly show that the rate of transphosphorylation is primarily contributed by Ca^2+^/CaM-bound subunits. However, more recent studies have found evidence that high kinase concentration enables subunit exchange and transphosphorylation between subunits within a holoenzyme can occur in the absence of Ca^2+^/CaM but on a much slower time course (406). It should also be considered that in vivo ambient Ca^2+^/CaM levels, although not sufficient to significantly activate the enzyme, might allow transient binding of Ca^2+^/CaM that is permissive for intersubunit phosphorylation within a holoenzyme at T286. Consistent with this is the finding that a high frequency of short exposures to Ca^2+^ is necessary for an effective rate of autophosphorylation of naive CaMKII in vitro but after a far lower frequency is adequate for autophosphorylation of a kinase with sufficient prior autophosphorylation (69).

Recent work (81) has proposed an additional mechanism for autophosphorylation of CaMKII that could support propagation of its autophosphorylated state. The authors show that in the presence of Ca^2+^/CaM mixing wt holoenzymes with a kinase-dead holoenzyme results in phosphorylation of the kinase-dead CaMKII. This could occur by the previously demonstrated subunit exchange, but it appears that in these experiments it was interholoenzyme phosphorylation rather than subunit exchange. Based on a series of experiments including mass photometry, cross-linking mass spectrometry and single-molecule TIRF microscopy, the authors fail to find support for exchange of subunits between holoenzymes. A key finding is that this phosphorylation was not blocked by prior cross-linking of the hub domains to their parent holoenzyme, i.e., holoenzymes with curtailed dimer shedding and subunit exchange. Although the data are convincing, it remains unclear why previous biochemical studies (15, 82, 406, 411, 412) failed to detect interholoenzyme autophosphorylation even when holoenzymes were incubated at high concentrations and standard assay temperature (411, 412). It is interesting to note that CaMKIIα, but not CaMKIIβ, can self-associate, at least at reduced pH or high concentration and this requires displacement of the autoinhibitory domain (133–135). Such an interaction could form the basis for interholoenzyme phosphorylation. Remaining to be demonstrated, if this mechanism is to propagate CaMKII activity, is that an inactive holoenzyme can be phosphorylated on T286 in the absence of Ca^2+^/CaM. This is what was demonstrated in the studies claiming to exhibit subunit exchange (406). The precise reasons for the difference in results remain to be sorted out.

It should be emphasized that the experiments of Stratton et al. (406) demonstrate that active holoenzymes (T286D) can phosphorylate wt holoenzymes in the absence of Ca^2+^/CaM. Although it can be argued as to whether this occurs through interholoenzyme phosphorylation or through subunit exchange, this finding shows that phosphorylation of inactive holoenzymes occurs (not bound Ca^2+^/CaM), thus establishing a phosphorylation event for maintaining CaMKII activity in the face of protein turnover.

The attractiveness of the subunit exchange model is that it confines the propagation of the signal to those holoenzymes that were originally activated. The interholoenzyme model runs the risk of runaway propagation of the signal to naive holoenzymes, thus further spreading the active state. To prevent the loss of synapse specificity of LTP, one needs to have a mechanism that prevents this spread from occurring. Phosphatases are an obvious candidate (see sect. 9.2.3). PP1 is the likely phosphatase for such a role, as it is concentrated in the PSD, whereas PP2A is abundant in the cytoplasm (64, 385, 386) and CaMKII in the PSD is preferentially dephosphorylated by PP1 (385). Thus, it seems reasonable that activated CaMKII that diffuses from the PSD into the spine cytoplasm would quickly be dephosphorylated.

The notion that CaMKII activity can survive protein turnover has recently been tested in hippocampal slice cultures, which can be maintained for many weeks, well beyond the complete turnover of CaMKII (413). These experiments took advantage of constitutive Ca^2+^-independent CaMKII activity (see sect. 8), which is maintained in slice cultures. Persistent CaMKII activity, in the absence of Ca^2+^ stimulation, remains stable over a 2-wk period. These results suggest that the nascent CaMKII protein present at 2 wk acquired its activity from preexisting active CaMKII molecules, transferring their activity to newly synthesized CaMKII molecules and thus maintaining the memory in the face of protein turnover.

How might subunit exchange or interholoenzyme phosphorylation occur at synapses? We know that for CaMKII to exert its effect it must be bound to GluN2B. The formation of CaMKII-GluN2B complexes generates a condensate that can impact other PSD properties, but this kinase-receptor association is not irreversible. It is likely that individual CaMKII holoenzymes can move into and out of the condensates, but as long as a net threshold level of these complexes remains the condensate persists. Movement of kinase in and out can provide the basis for subunit exchange or interholoenzyme phosphorylation. In such a scenario, there is no need for the condensate to be recycled; it is the contents of the condensate that would turn over. Furthermore, it seems reasonable that the CaMKII cluster will be anchored in the PSD by more than one GluN2B, in which case the turnover of NMDARs can occur while preserving the anchoring of CaMKII to the PSD.

Finally, it is of interest to consider the evidence that CaMKII activity can withstand protein turnover to structural changes associated with LTP. LTP is associated with a long-lasting increase in spine size (247, 248), including a late increase in the size of the PSD (56, 213, 414, 415) and additionally a slow increase in the size of the presynaptic bouton (213, 414). Protein synthesis is also proposed to be required for “late” LTP (55–57) (see sect. 3.3). One might expect that these late changes would be more stable, resisting reversal and, perhaps, ultimately independent of CaMKII signaling. Perhaps even more problematic is the report that a substantial portion of dendritic spines on CA1 pyramidal cells turn over within a few weeks (416, 417). How one can maintain a CaMKII memory trace in the face of such impermanence of dendritic spines remains a mystery.

## 11. CaMKII AND HIPPOCAMPAL PLACE FIELDS

**Figure 12.**
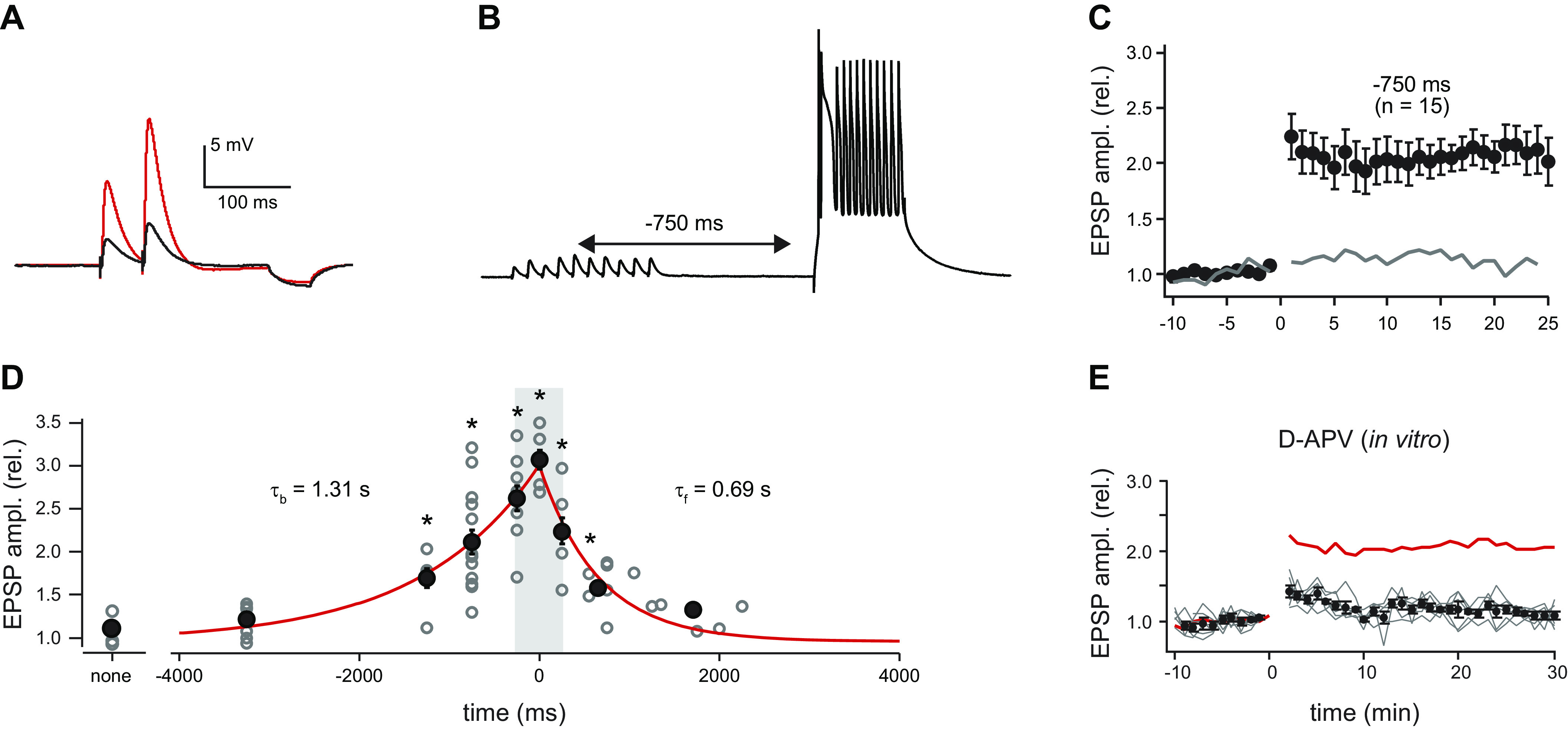
Behavioral timescale synaptic plasticity. *A*: excitatory postsynaptic potentials (EPSPs) used to determine synaptic strength (50-ms interval). Black trace is average baseline EPSP; red trace is average postpairing EPSP. Hyperpolarization following EPSPs from 50-ms, –25-pA current injection used to determine input resistance (*R*_in_). *B*: membrane potential (*V*_m_) trace showing representative induction protocol with 10 synaptic stimuli (20 Hz) followed by plateau potential (300-ms current injection). *C*: average EPSP amplitude (normalized to baseline; ±SE) for population of neurons that received the indicated induction protocol. Induction (5 pairings) at 0 min. Gray line is average EPSP amplitude for synaptic stimulation alone. rel., Relative. *D*: plot of postinduction EPSP amplitude normalized to baseline vs. the induction interval time for the entire population of neurons. Open gray symbols are individual neurons; black symbols are means. Tau backward (t_b_) from exponential fit of data ranging from 0 to –3,250 ms (red line projecting to negative times). Tau forward (t_f_) from exponential fit of data ranging from 0 to +2,000 ms (red line projecting to positive times). Synaptic stimulation alone (no pairing interval, “none”) not included in exponential fits. See supplementary methods in Ref. 104 for means and *P* values. *E*: effect of 20 µM d-2-amino-5-phosphonopentanoic acid (d-APV; *left*). Average EPSP amplitude (normalized to baseline; ±SE) for population of neurons that received –750-ms interval induction protocol. Red line is mean for control (from *C*). Gray lines are individual neurons. Modified from Ref. 104., with permission from *Science*.

An intriguing long-established property of the hippocampus is its ability to form place fields in which pyramidal cells become active when an animal enters a particular place in its environment. The mechanism(s) underlying the formation and stability of place fields is complex. However, pharmacological blockade of NMDARs prevents the stabilization of new place fields (418, 419), and the genetic deletion of GluN1 in the CA1 region of the hippocampus decreases the spatial specificity of place fields (420). Furthermore, studies in knockin mice containing the T286A mutation of CaMKII show the importance of CaMKII in spatial selectivity (421, 422). The possible role of LTP in the formation and maintenance of place fields has been reviewed (423). A series of elegant studies (104, 106, 424, 425) have discovered a new form of plasticity referred to as behavioral timescale synaptic plasticity (BTSP). Input to CA1 from entorhinal cortex provides an instructive signal in the form of large Ca^2+^-dependent dendritic plateau potentials (424, 426). These plateau potentials on their own do not change synaptic strength. However, when these plateau potentials are coincident with or are preceded by several hundreds of milliseconds by a series of subthreshold excitatory postsynaptic potentials (EPSPs), these EPSPs are potentiated. BTSP has been reproduced in slices (104) (FIGURE 12, *A–C*). In this case the plateau potential is induced by strong postsynaptic depolarization. The potentiation of EPSPs can occur with intervals of up to 2 s between the synaptic responses and the plateau potential (FIGURE 12*D*). What is the nature of the seconds-long trace left by the subthreshold EPSPs (referred to as an eligibility trace)? Pharmacological blockade of NMDARs blocks BTSP (FIGURE 12*E*), suggesting that the eligibility trace requires NMDAR activation. But how can a subthreshold EPSP activate NMDARs? Recent biophysical experiments on spines provide some insight. Because of the high spine neck resistance (∼1 GΩ) it is calculated that an unpotentiated EPSP can approximate 25 mV (185, 186). Such a depolarization is expected to unblock some NMDAR and result in a small influx of Ca^2+^, which by itself would not be sufficient to alter synaptic strength. A logical mechanism for the eligibility trace is partial activation of CaMKII. This would not be enough to alter synaptic transmission, and the CaMKII would quickly be dephosphorylated (see sect. 3.1). The plateau potential would provide a sufficient rise in Ca^2+^ to fully activate CaMKII. In this scenario there is coincidence between the subthreshold partially activated CaMKII and the voltage-dependent Ca^2+^ entry during the plateau potential. Another possibility is that there is a 2- to 3-s-long NMDA-mediated metabotropic action (399, 427), which when paired with the Ca^2+^-dependent plateau potential strengthens the synapse. The finding that strong hyperpolarization before the plateau potential blunts the potentiation (103) favors the former but does not rule out the latter. In summary, BTSP is a homosynaptic form of LTP that requires NMDAR activation. However, unlike classical forms of LTP where NMDARs are gated by coincident depolarization provided by either postsynaptic spikes or spatiotemporal integration of other synaptic inputs, it appears that BTSP requires only that NMDARs be gated by local spine depolarization. Rather, the coincidence is between an NMDA-induced seconds-long eligibility trace (CaMKII?) and the large postsynaptic depolarization provided by the dendritic Ca^2+^ spike.

## 12. HUMAN GENETIC MUTATIONS IN CaMKII

Several dozen human mutations in CaMKII have been identified, and their molecular characteristics support many of the biochemical behaviors of CaMKII described above. However, none blocks generation of constitutive activity, comparable to the experimental Thr286A mutation, that would enable a test of Thr286 phosphorylation and constitutive CaMKII activity in human memory (428–430). The mutations were found by exome sequencing of infants with mild to severe developmental and intellectual disabilities. These gain-of-function and loss-of-function mutations are consistent with our understanding of the autoinhibitory segment, the activation by Ca^2+^/CaM, the autophosphorylation generating autonomy, and even CaM trapping. They also suggest that neurodevelopmental effects are very sensitive to even small changes in biochemical parameters, particularly mutations that increase basal autonomy. Several of the CaMKII mutants have undergone detailed biochemical characterization. Many of the mutations decrease interactions between the kinase surface and the autoinhibitory segment previously shown to increase basal activity that is normally quite low. These in turn increase autophosphorylation of T286 (or T287 in some isoforms) that further increases constitutive activity (429, 430). One CaMKIIα mutant, E183V, is near the kinase surface, interacting with the autoinhibitory segment, and leads to reduced phosphorylation of external substrates as well as itself (T286 autophosphorylation) (428). Consistent with a surface groove accommodating both substrates and anchoring proteins, it also exhibits reduced interactions with GluN2B and other interacting proteins. Expression of the mutant in neurons decreases spine density and synaptic transmission. In one case, a mutation in the hub domain (H477Y in CaMKIIα) disrupts the assembly of CaMKII into multimeric holoenzymes, leading to severe intellectual disabilities and seizures (431).

To date, a mutation of T286 to a nonphosphorylated neutral amino acid has not been found, so a direct human link between T286 autophosphorylation and its potential step in memory described in this review cannot be made. Of course, the pathophysiological repertoire seen in human subjects is complicated by effects of the kinase during growth and development of the brain. Nevertheless, there are additional interesting findings. For example, although we normally consider only the α- and β-isoforms when discussing brain function, the others are not absent but are just not present at the very high levels of the α- and β-isoforms. Thus, it is interesting that several subjects with a R292P mutant in CaMKIIγ have been identified with severe intellectual disability. The mutation is in the autoregulatory segment, and it is likely that the disruption of its conformation by the presence of a proline reduces autoinhibition and produces increased autonomy that has a gain-of-function effect in the cytosol (432). The same mutation that increases autonomous activity produces a loss of function in shuttling Ca^2+^/CaM from synapse to nucleus (433). R292 is near residues that CaM interacts with after autophosphorylation to produce CaM trapping. The structural disruption caused by R292P greatly reduces CaM trapping and thereby blocks the ability of autonomous CaMKIIγ to carry the cargo of CaM that it normally releases in the nucleus to stimulate CREB phosphorylation and gene expression.

## 13. CONCLUSIONS

### 13.1. CaMKII and Its Activation

**Figure 13.**
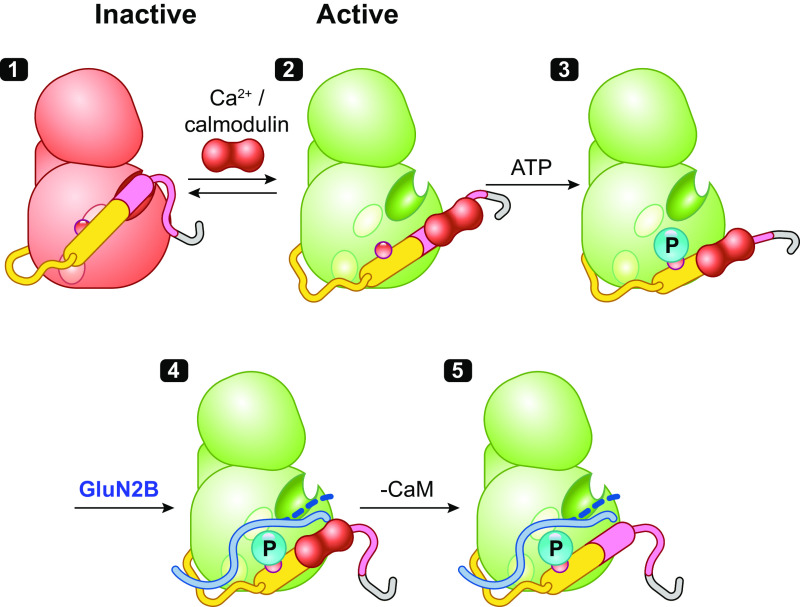
Proposed sequence of events underlying activation of Ca^2+^/calmodulin-dependent protein kinase II (CaMKII). We next turn to how the CaMKII-GluN2B complex enhances synaptic transmission. Three models are discussed in this review (see FIGURE 7). The first model (receptor-centric model) posits that CaMKII phosphorylates the COOH-terminal domain (CTD) of α-amino-3-hydroxy-5-methyl-4-isoxazolepropionic acid receptors (AMPARs) and/or transmembrane AMPAR-regulatory protein (TARPs), which are then captured in the postsynaptic density (PSD). The second model (PSD-centric model) proposes that CaMKII modifies component(s) in the PSD creating slots, perhaps unmasking PSD-95 and then allowing the capture of passively diffusing unaltered AMPAR/TARPs. The third model (vesicle-centric model) proposes that CaMKII initiates, by mechanisms that have yet to be fully elucidated, exocytosis of AMPAR-containing vesicles. Although there is an undeniable bias in this review for the PSD-centric model, these models are certainly not mutually exclusive. It should be pointed out that, although it is well established that CaMKII phosphorylates numerous synaptic proteins (66, 434–437), as reviewed here, despite intensive research an essential role for CaMKII phosphorylation of many of these targets largely remains elusive. Perhaps these negative results are telling us something.

There were periods during the past 40 years when the importance of CaMKII in various aspects of LTP was severely challenged. However, there is now little doubt that NMDAR activation, which raises resting Ca^2+^ (∼50 nM) to levels high enough to activate the low-affinity CaMKII (half maximal = 1.6 µM), is the primary initial step in LTP. Not only is it critical for inducing LTP, but evidence now strongly supports the hypothesis that CaMKII is also critical for maintaining LTP (memory). FIGURE 13 summarizes the sequence of events that follows the rise in spine Ca^2+^ initiated by NMDAR activation. For simplicity, we illustrate a single subunit. Ca^2+^ binds to CaM, which, in turn, binds to CaMKII (FIGURE 13, *1* to *2*). The peeling away of the regulatory domain caused by the binding of Ca^2+^/CaM results in the phosphorylation of T286 (FIGURE 13, *2* to *3*) and the translocation of CaMKII to the PSD, where it binds to the CTD of GluN2B (FIGURE 13, *3* to *4*). After the Ca^2+^ level returns to resting values, the CaMKII-GluN2B complex remains intact (FIGURE 13, *4* to *5*). This CaMKII-GluN2B complex is critical for the synaptic actions of CaMKII. The status of T286 phosphorylation when CaMKII is bound to GluN2B is hotly debated. Although T286 phosphorylation is not required for CaMKII’s binding to GluN2B, it is likely that autophosphorylation would precede this binding and that the kinase would remain phosphorylated, barring a depotentiating stimulus. As reviewed above, there is recent evidence that T286 phosphorylation is required for maintaining the stability of the CaMKII-GluN2B complex.

### 13.2. Reorganization of PSD during LTP

**Figure 14.**
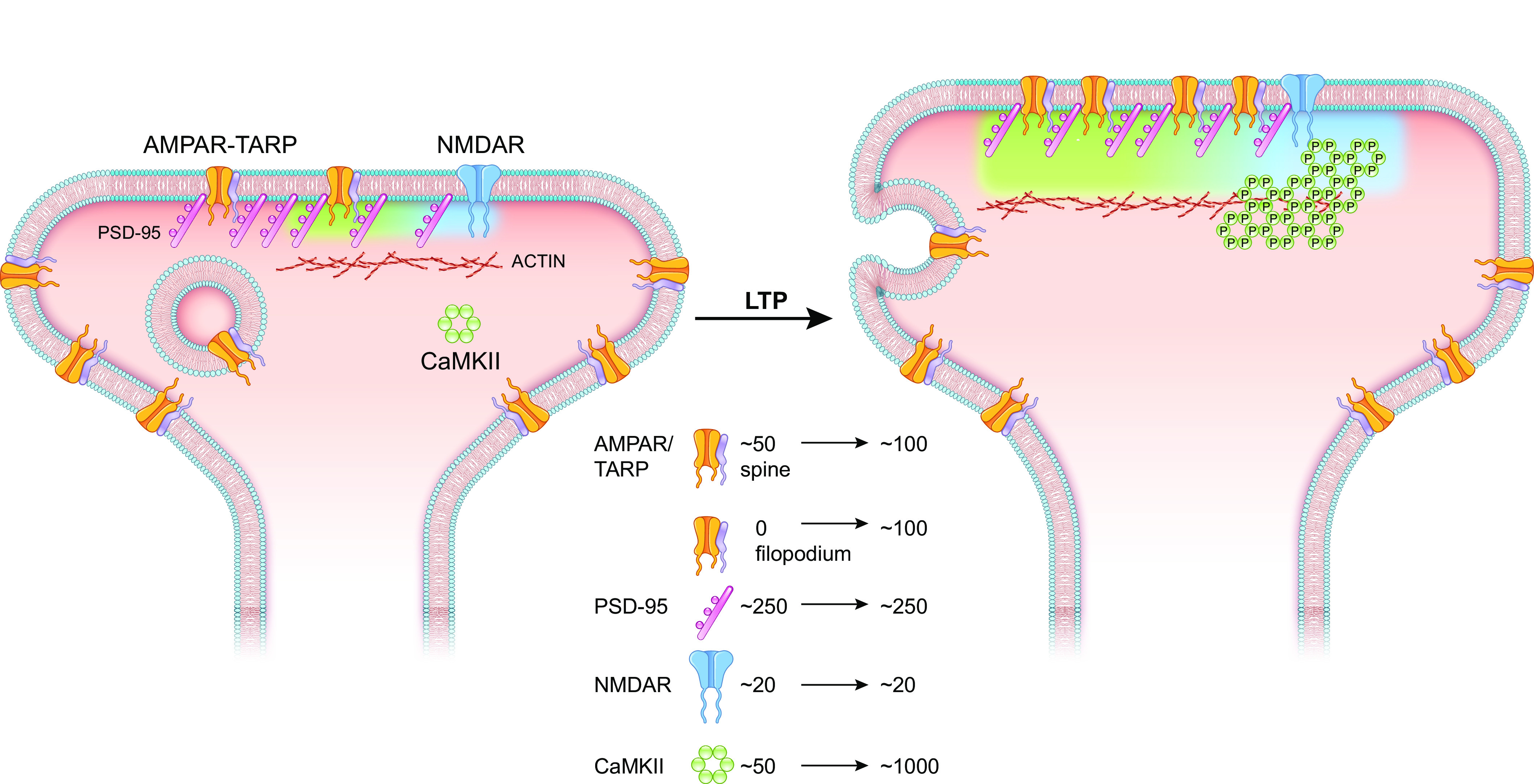
The proposed sequence of events underlying long-term potentiation (LTP). This figure focuses on spine synapses, but a substantial number of synapses are formed on filopodia and assumed to be like spine synapses but are lacking α-amino-3-hydroxy-5-methyl-4-isoxazolepropionic acid receptor (AMPAR)/transmembrane AMPAR-regulatory proteins (TARPs). The diagram does not accurately portray the stoichiometry of the various proteins, and actin is distributed throughout the spine. In the resting state the number of PSD-95 molecules outnumbers the AMPAR/TARPs. An LTP-inducing stimulus activates *N*-methyl-d-aspartate receptors (NMDARs), causing a rise in spine Ca^2+^. Ca^2+^ via binding to calmodulin (CaM) activates Ca^2+^/CaM-dependent protein kinase II (CaMKII). This results in self-association and the binding to the COOH-terminal domain (CTD) of GluN2B. The CaMKII-GluN2B complex is proposed to undergo phase separation (light blue shaded area), which results in a distinct phase separation of AMPAR/TARPs and PSD-95 (light green shaded area). This may be the mechanism enabling PSD-95 to capture AMPARs that normally diffuse into and out of the synapse. This process may be augmented by the exocytosis of AMPAR-containing vesicles. Accompanying these changes is an enlargement of the spine. Most important is a widening of the spine neck, which will have consequences for both chemical and electrical compartmentalization.

Before considering the changes that occur during LTP, it is important to review the stoichiometry of the key proteins in the PSD (FIGURE 14). The number of AMPARs at dendritic spine synapses is highly variable but averages roughly 50 (143, 438, 439), and this number strongly correlates with the size of the PSD. This number presumably also applies for TARPs. Recent work has shown that synapses with few, if any, AMPARs, referred to as silent synapses (238, 239), form a distinct anatomical class referred to as filopodial synapses, in contrast to classical spine synapses (240). The number of NMDARs at spine synapses and perhaps filopodia synapses is much less variable, averaging roughly 20, and this number is largely independent of PSD size (143, 438, 439). The number of PSD-95 molecules is roughly 200–300 (65), and the number of CaMKII holoenzymes is estimated to be roughly 80 (65). Most of these molecules are in the pallium, just beneath the core of the PSD and not close enough to be bound to GluN2B (139). Given that CaMKII must be bound to the NMDAR for its synaptic action, it is likely that most of these molecules represent a reserve inactive pool. What can we conclude from these stoichiometries? It is generally accepted that AMPARs are abundant on the extrasynaptic membrane and freely diffuse into and out of the PSD (329). It is also agreed that the synaptic capture of AMPARs requires the PDZ domain interaction between TARPs and PSD-95 (275, 279, 294, 440). Yet there are far more PSD-95 molecules in the PSD compared to AMPARs. These findings suggest that a substantial number of PSD-95 molecules are not available for capturing AMPARs.

In what way does the PSD protein stoichiometry change after LTP (FIGURE 14)? We focus on the first hour since this has received the vast amount of research. The events that occur during LTP can be roughly divided into three components: *1*) the formation of the CaMKII-GluN2B complex, *2*) the recruitment of AMPARs, and *3*) structural changes. The first component involves the NMDAR activation rise in Ca^2+^, which binds to CaM and activates CaMKII. Activated CaMKII binds to the COOH terminus of GluN2B and is postulated to undergo self-association and promote LLPS. The number of CaMKII molecules bound to the PSD in a fully activated synapse is estimated to be 270 (65). The number of NMDARs does not change, since constitutively active CaMKII does not affect the NMDAR EPSC (130–132) and LTP is expressed primarily on the AMPAR (19). Thus, bound CaMKII far outnumbers the NMDARs, indicating that the large clusters are anchored to the PSD by the minority of subunits that directly bind to the NMDARs (FIGURE 14).

Such a macromolecular complex has many features that foster information storage. It allows for the dynamic binding and unbinding of components while maintaining the overall complex. It could shield the active CaMKII from phosphatases. It provides a substrate for propagating autophosphorylated CaMKII, which turns over in ∼2 wk, either through subunit exchange of inactive subunits into active holoenyzmes or by interholoenyzme phosphorylation. In addition, such a complex can accommodate the turnover of NMDARs, which also occurs in ∼2 wk. The driver of potentiation is not dependent on individual CaMKII-GluN2B complexes but on dozens of such complexes in a self-propagating network. This provides a margin of safety in information retention, as individual dephosphorylation or protein turnover events do not depotentiate the synapse. It is positioned close to the postsynaptic membrane and PSD via the tether to NMDARs and provides an excess of spare docking sites for NMDARs on the CaMKII holoenzyme network.

The second component of LTP is the trafficking of AMPARs to the PSD. This discussion is based on studies of spine synapses. Much less is known about filopodial synapses, which are the anatomical substrate for silent synapses, which lack AMPAR/TARPs (240). Although there is no change in the number of PSD-95 molecules (56), saturating LTP results in a two- to threefold enhancement of the AMPAR EPSC, as does the expression of constitutively active CaMKII. Thus, the number of AMPARs in the PSD increases two- to threefold. There are three proposed sources for these AMPARs. First, AMPARs in the surrounding extrasynaptic membrane can diffuse into the PSD. We feel the evidence is strongest for this model. Second, it is proposed that AMPARs are exocytosed near the PSD. Although exocytosis is required for LTP, there is still debate over whether the cargo is AMPARs or some other critical molecule. The third source involves the redistribution of PSD AMPARs. As reviewed in sect. 7.1, the PSD is composed of nanocolumns, such that AMPARs are clustered across from presynaptic release sites. In this model dispersed AMPARs in the PSD, which do not “see” activating levels of glutamate, redistribute to these nanocolumns. Importantly, these three models are not mutually exclusive.

The remaining key question is how the formation of the CaMKII-GluN2B complex initiates the trafficking of AMPARs. The recent findings of Hosokawa et al. (323) show that the CaMKII-GluN2B undergoes LLPS. Most intriguingly, this orchestrates a phase-in-phase condensation of AMPAR/TARPs, PSD-95, and neuroligin1, largely independent of phosphorylation, although T286 phosphorylation is required for the maintenance of the condensates. Such a CaMKII-GluN2B hub may be the driver of increased AMPAR/TARPs that is the basis for LTP expression. Presumably in this LLPS reorganization, PSD-95 molecules previously not available for AMPAR/TARPs become unmasked. These results certainly do not exclude a protein kinase role for CaMKII in LTP. Indeed, there is compelling evidence that CaMKII, via phosphorylation, initiates a series of actin-based cytoskeletal rearrangements in the spine (32, 312–314). Admittedly, the proposed role of LLPS in LTP is speculative. Although LLPS has been demonstrated in solution and has many attractive features, it remains to be determined whether it occurs with membrane proteins such as TARPs reconstituted in lipid bilayers next to the condensates and ultimately in the PSD. If so, how might the cytoskeletal rearrangements facilitate assembly of the critical players in LTP?

The third component of LTP is structural. Parallel to the electrophysiological changes during LTP is an enlargement of the spine and a widening of the spine neck (247, 248). These morphological changes involve actin-based cytoskeletal remodeling (see sect. 7.4.3) and will impact both biochemical and electrical compartmentalization. Actin is largely excluded from the core of the PSD (66, 322), and evidence suggests that, at least for the first hour, the changes in spine size are not accompanied by changes in the size of the PSD (56). These findings might suggest a dissociation of structural and physiological LTP. However, blocking actin remodeling blocks both sLTP (247) and LTP (310). Thus, it remains unclear how actin polymerization communicates with core elements of the PSD. Might actin participate in the LLPS initiated by CaMKII-GluN2B binding?

### 13.3. CaMKII and Information Storage

Based on our current knowledge of CaMKII and LTP, it is interesting to revisit the prescient theoretical contributions of Crick (7) and Lisman (8, 70). As discussed above, they proposed a molecular model for information storage that could withstand molecular turnover. Their model has two requirements. The first requirement is a multimeric protein in which subunits can phosphorylate one another (at a site generating autonomy). As summarized in this review, CaMKII fulfills this requirement beautifully, and recent evidence suggests that this autonomy is responsible for maintaining LTP. The second requirement is that subunits from newly synthesized naive holoenzymes exchange into active holoenzymes. The recent work from Kuriyan and collaborators (406) nicely fulfills this second requirement, although there may be an alternative mechanism of interholoenzyme phosphorylation (81). The latter mechanism requires constraints to guard against the exponential spread of such a mechanism.

Our comprehensive review of the properties of CaMKII and those of LTP emphasizes how well the properties of CaMKII can explain virtually all the physiological properties of LTP, including synaptic memory. However, although there is considerable evidence linking LTP to behavior memory (45, 441–443), more evidence is needed to solidify this link. For instance, recent experiments (46) using the viral expression of the catalytically dead CaMKII K42M mutant, which acts in a dominant-negative manner (444), have found that transiently silencing CaMKII in mice after learning a behavior erases the memory (46). Further experiments along these lines would greatly strengthen the linkage between synaptic physiology and behavior. These are exciting times in the field of synaptic plasticity. We are within reach of a detailed molecular understanding of the synaptic changes that are triggered by experiences and then retained as a memory of that experience.

## GRANTS

R.A.N. is supported by National Institute of Mental Health Grant R01MH117139.

## DISCLOSURES

No conflicts of interest, financial or otherwise, are declared by the authors.

## AUTHOR CONTRIBUTIONS

R.A.N. drafted manuscript; R.A.N. and H.S. edited and revised manuscript; H.S. approved final version of manuscript.
